# Perspectives on terahertz honey bee sensing

**DOI:** 10.1038/s41598-025-91630-8

**Published:** 2025-03-27

**Authors:** Andreas Prokscha, Fawad Sheikh, Mandana Jalali, Pieterjan De Boose, Eline De Borre, Vera Jeladze, Felipe Oliveira Ribas, David Toribio Carvajal, Jan Taro Svejda, Tobias Kubiczek, Basem Aqlan, Pooya Alibeigloo, Enes Mutlu, Jonas Watermann, Jonathan Abts, Robin Kress, Christian Preuss, Simone Clochiatti, Livia Wiedau, Nils G. Weimann, Jan C. Balzer, Arno Thielens, Thomas Kaiser, Daniel Erni

**Affiliations:** 1 Institute of Digital Signal Processing (DSV), University of Duisburg-Essen (UDE), Duisburg, 47057 Germany; 2 General and Theoretical Electrical Engineering (ATE), University of Duisburg-Essen (UDE), and Center for Nanointegration Duisburg-Essen (CENIDE), Duisburg, 47048 Germany; 3 Department of Information Technology, Ghent University, Ghent, 9052 Belgium; 4 Department of Biocybernetics, Institute of Cybernetics of the Georgian Technical University, Tbilisi, 0186 Georgia; 5 Department of Information Technology, Georgian National University SEU, Tbilisi, 0144 Georgia; 6 Chair of Communication Systems (NTS), University of Duisburg-Essen (UDE), Duisburg, 47057 Germany; 7 Department Components for High-Frequency Electronics (BHE), University of Duisburg-Essen (UDE), Duisburg, 47057 Germany; 8 Chair of Manufacturing Technology, University of Duisburg-Essen (UDE), Duisburg, 47057 Germany; 9 Department Components for High-Frequency Electronics (BHE), University of Duisburg-Essen (UDE), and Center for Nanointegration Duisburg-Essen (CENIDE), Duisburg, 47057 Germany; 10 Photonics Initiative, Advanced Science and Research Center, The Graduate Center of the City University of New York, New York, 10030 USA

**Keywords:** Environmental sciences, Electrical and electronic engineering

## Abstract

Terahertz (THz) technology provides precise monitoring capabilities in dynamic environments, offering unique insights into insect habitats. Our study focuses on environmental monitoring of European honey bees (Apis mellifera) through a combination of measurements and simulations. Initially, the dielectric material properties of honey bee body parts are characterized across the spectral range of 1–500 GHz to collect heterogeneous empirical data. To extend the study, honey bee mockups made from polyamide 12 (PA12) and epoxy resin are employed and validated as effective substitutes for real bees through comparative scattering analyses. The research further explores radar cross-section (RCS), imaging, and spectral properties using advanced THz technologies, including resonant tunneling diodes (RTDs) operating at 250 GHz and THz time-domain spectroscopy (THz-TDS) for frequencies exceeding 250 GHz. High-resolution imaging, utilizing a 450 GHz bandwidth, captures intricate anatomical features of both real and 3D-printed bees, showcasing the potential of THz technology for detailed environmental monitoring. Finally, simulations at 300 GHz assess the dosimetry and feasibility of non-invasive, continuous monitoring approaches based on the heterogeneous honey bee model.

Information provided by wireless communication technologies can aid in detecting and interpreting biotopes impacted by the effects of climate change. In recent years, THz communications and sensing systems operating at 0.1–10 THz^1^ have become an increasingly popular area of research for biosecurity^2^, accompanied by continuous environmental monitoring with precise localization and material characterization capabilities. Concluding the research in wireless communication communality, this THz region, located between millimeter wave (mmWave) and near-infrared area, is of great interest comprising non-destructive, non-ionizing, and non-invasive characteristics^3,4^. However, molecular resonances result in attenuation in the THz region, making it essential to identify frequency bands with minimal losses for wireless communication technologies and sensing^5,6^. The miniaturization capability of THz antennas, owing to small resonating wavelengths^7^, makes them suitable for implementing sensors that receive and transmit, enabling comprehensive monitoring for plants or insects, incorporating the ecological interactions with their environment and the behavior in habitats^2^. At the same time, the deployment of 5G and future 6G mobile communication systems, coupled with the potential impact of electromagnetic (EM) radiation, has raised concerns about their effects on insects, particularly honey bees^8^. Insects, which are essential for ecosystem functions like pollination, have been facing declining populations due to factors such as pesticide use and habitat loss^9^. Especially, insects such as European honey bees (*Apis mellifera*) interacting with plants in pollination symbiosis are of tremendous ecological and economical importance^10^. Up to 84% of food provisions related to global food crop production depend on pollination activities of animals^11^, which is similar to the number of flowering plants reproducing due to this plant-pollinator interaction^12^. Given the critical role of pollinators in sustaining both natural ecosystems and human society, the need for precise and continuous monitoring in dynamic environments is undeniable. Accurate insect monitoring is crucial for understanding the changing conditions of these habitats^2^. Effective monitoring technologies must meet diverse requirements, including minimizing disruption to natural ecosystems and moving beyond traditional methods that rely on studying deceased insects, labor-intensive sorting, and specialist-dependent species identification^13^. Furthermore, these technologies should be non-invasive, cost-effective, field-tested, user-friendly for a single operator, and capable of large-scale, real-time monitoring^13–15^. Consequently, various aspects must be taken into account when evaluating THz technology, which serves as the motivation for presenting multiple perspectives in this work. When evaluating dosimetry, it is essential to consider that the interaction between EM fields and dielectric materials can cause heating^16,17^. As with any type of exposure to EM radiation, it is important to be mindful of the duration and intensity of exposure, and to use protective measures when necessary, especially when living organisms are involved like humans, plants or animals. This EM induced exposure is frequency and object size dependent^18^. Thus, the introduction of high-frequency EM radiation into the environment has led to questions about whether it might affect insects, especially considering their small size and the wavelengths in the multi-gigahertz (GHz) to THz range. Noteworthy to mention, this phenomenon does not occur uniformly throughout its body, given its multi-component anatomy with varying lengths and electrical properties. Pioneering studies in the 0.6–120 GHz frequency range have distinguished between different honey bee types, revealing increased EM radiation absorption, particularly at 5G frequencies and beyond^19,20^.

**Fig. 1:**
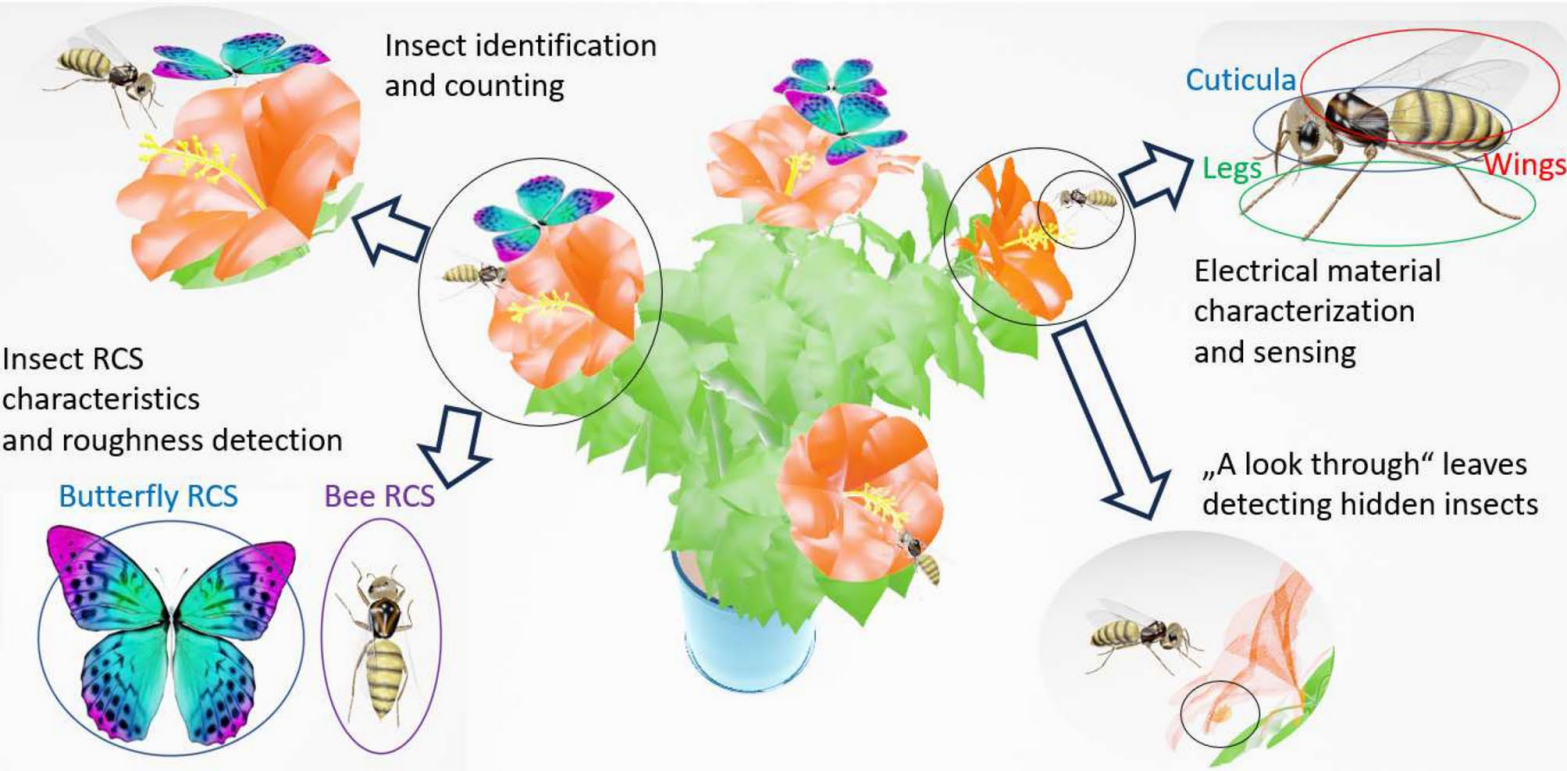
Proposed application area: Insect environmental monitoring with THz technologies.

Another important aspect is the reliable monitoring of insects through their spectral fingerprints. In a preliminary study, a mmWave radar was employed to monitor the scattered power of an 8 mm-long ladybug (Coccinellidae)^2^. The primary objective was to develop a technique for using THz radar to monitor even smaller insects like aphids. Although the experiment focused on a relatively larger ladybug, it successfully demonstrated the feasibility of using radar technology, even at mmWave frequencies, to track the movement of smaller insects. The results indicated the potential for monitoring smaller insects, such as aphids, using THz radar at higher frequencies based on the visible and distinguishable movement of the ladybug observed in the subtraction images. The RCS is a vital parameter in radar systems, quantifying the amount of EM energy reflected from an object at a specific angle. In particular state-of-the-art radar entomology is currently considering small-scale radar systems mostly in the X-band (having also systems up to the K$_{\rm a}$-band^21^) for the model-based identification of morphological insect parameters as part of the essential biodiversity variables (EBVs)^22^. Such systems operate in an intermediate range between several meters up to kilometers extracting aggregated behavioral and biological parameters of migrating insects, such as flight altitude, population density, displacement direction and speed, head orientation (e.g., determined through polarization pattern analysis), body mass, and wingbeat frequency. Nevertheless, these approaches have traditionally been considered unsuitable for biodiversity monitoring due to their difficulty in identifying insects uniquely. In order to increase taxonomic resolution multifrequency and fully polarized small-scale radar systems have been proposed in conjunction with machine learning (ML)-based classification^23^. In our case we are investigating continuous insect monitoring in close ecosystems of pollinators (e.g. bees) and pests interacting with model plants using broadband miniaturized THz radar systems^2^. This enables the correlation of rich RCS patterns with highly resolved physical details and morphological characteristics, accurately reflecting the shape and orientation of bees, thereby offering a novel approach to small-scale radar entomology. Traditional radar systems for insect RCS typically use frequencies in the 6 GHz to 12 GHz range, suitable for larger insects^24,25^. Recent radar advancements explore higher frequencies, such as 24 GHz and 77 GHz, to monitor smaller insects^26,27^. However, these high-frequency radars offer limited detail due to the insects’ small size. Researchers are thus exploring even higher frequencies beyond 100 GHz to detect and classify smaller objects more effectively, enabling the capture of finer details and distinctions, conducted with THz time-domain spectroscopy (THz-TDS) systems^28^. The choice of radar frequency is critical, particularly for small objects, where wavelength and object size are of comparable magnitude. The backscattering of EM waves by insects is primarily influenced by factors such as body roughness, angle of incidence, complex refractive index, and wavelength. At THz frequencies, the insect’s body roughness becomes more pronounced, resulting in significantly stronger backscatter effects^2^. Furthermore, the scattering behavior of a real honey bee demonstrated a correlation between its orientation and the polarization of THz waves^2^.

Figure 1 illustrates the envisioned THz-derived information for insect monitoring, including recognition based on anatomy, electrical properties, or RCS. Dynamic imaging challenges can be addressed by employing a multiple-input multiple-output (MIMO) radar system^2^, which leverages the huge bandwidth available in the THz spectrum while maintaining small system form factors for minimal environmental intervention. Sub-mm range and angular resolution enable the identification of small objects and provide information on their RCS or surface roughness. 3D THz images provide depth information, revealing not only the external appearance of objects but also their internal structures and layers. Additionally, material characterization can be achieved due to the frequency-dependent complex permittivity, which is essential for developing a THz database for multi-layer and multi-material object recognition and for creating efficient real-time THz material maps using THz transceivers^29^. Such THz data can be integrated with image data to provide a more reliable object description. Previous research has demonstrated the transparency of dielectric materials, such as fabrics^30^, and the identification of insect body parts in tea^31^, as well as THz penetration characteristics in leaves^32^. Such findings support the goal of systematic environmental monitoring, a challenge with currently established visual methods like human observation or camera-recorded imagery. This work provides a comprehensive examination of various aspects, including dosimetry, dielectric material characterization of honey bee parts, and imaging. In addition to the heterogeneous material characterization of honey bee parts, our measurements aim to investigate the RCS and scattered power using additively manufactured (AM) honey bee models made of PA12 and epoxy resin. It not only highlights spectral characteristics as distinctive features for reliable honey bee monitoring but also demonstrates similarities in the scattering behavior of the presented honey bee mockups compared to real honey bees. The THz-TDS system provides a bi-static setup for precise RCS estimation, unveiling bandwidth requirements directly related to achieving range resolution capable of resolving anatomical details of the bee at the sub-mm scale. Furthermore, to justify additively manufactured bee models as a suitable and ethically acceptable alternative to real honey bees, their similarities in scattering behavior are demonstrated in the context of THz imaging. The novel concept of RTD oscillator chips operating at 250 GHz reveals a correlation between the orientation of bee mockups and EM wave polarization, as previously reported for real bees^2^. This technology represents significant progress toward the ultimate goal of developing miniaturized THz systems^2^. These measurement campaigns are complemented by an extensive material characterization of various bee parts, highlighting an additional novelty in our work for the THz community. This is also reflected in recent literature surveys on the radio-frequency EM exposure of invertebrates commissioned by the EU^33^ and in particular by the Swiss Federal Office for the Environment BAFU^8^, where a considerable lack of reliable data and therefore a need for further research has been identified distinctly for the mmWave/THz frequency range. Typically, insects have been investigated previously assuming models with homogeneous dielectric properties throughout their entire bodies. This material characterization, specifically for wings, cuticula, inner parts, and the entire bee, significantly enhance the meaningfulness of EM simulations for predicting exposure in a honey bee’s body. Furthermore, this study explores several novel facets of insect-related monitoring within the sub-THz frequency range. Firstly, it delves into the RCS of AM honey bee models, validating prior research conducted with real bees^2^, leveraging cutting-edge THz systems, and opening avenues for miniaturized THz radar-based measurements. Additionally, it introduces, for the first time, a comprehensive material characterization of European honey bees, establishing a heterogeneous body model. Furthermore, it investigates RF exposure across various parts of the honey bee anatomy in sub-THz frequencies, under both near-field and far-field conditions.

## Methods

### The insect is a European honey bee (Apis mellifera)

This bee, originated in Europe is the most common honey bee. It should be noted that our approach to the European honey bee is an ongoing multi-staged process using correspondingly prepared dead bees in our experiments together with precise 3D-printed bee mockups, all of which is supported by numerical modeling based on digital twins, as dead bees with “real bee characteristics” do not exist due to fast dry out respective decomposition and experiments with living bees are not feasible.

### Dielectric properties

**Fig. 2:**
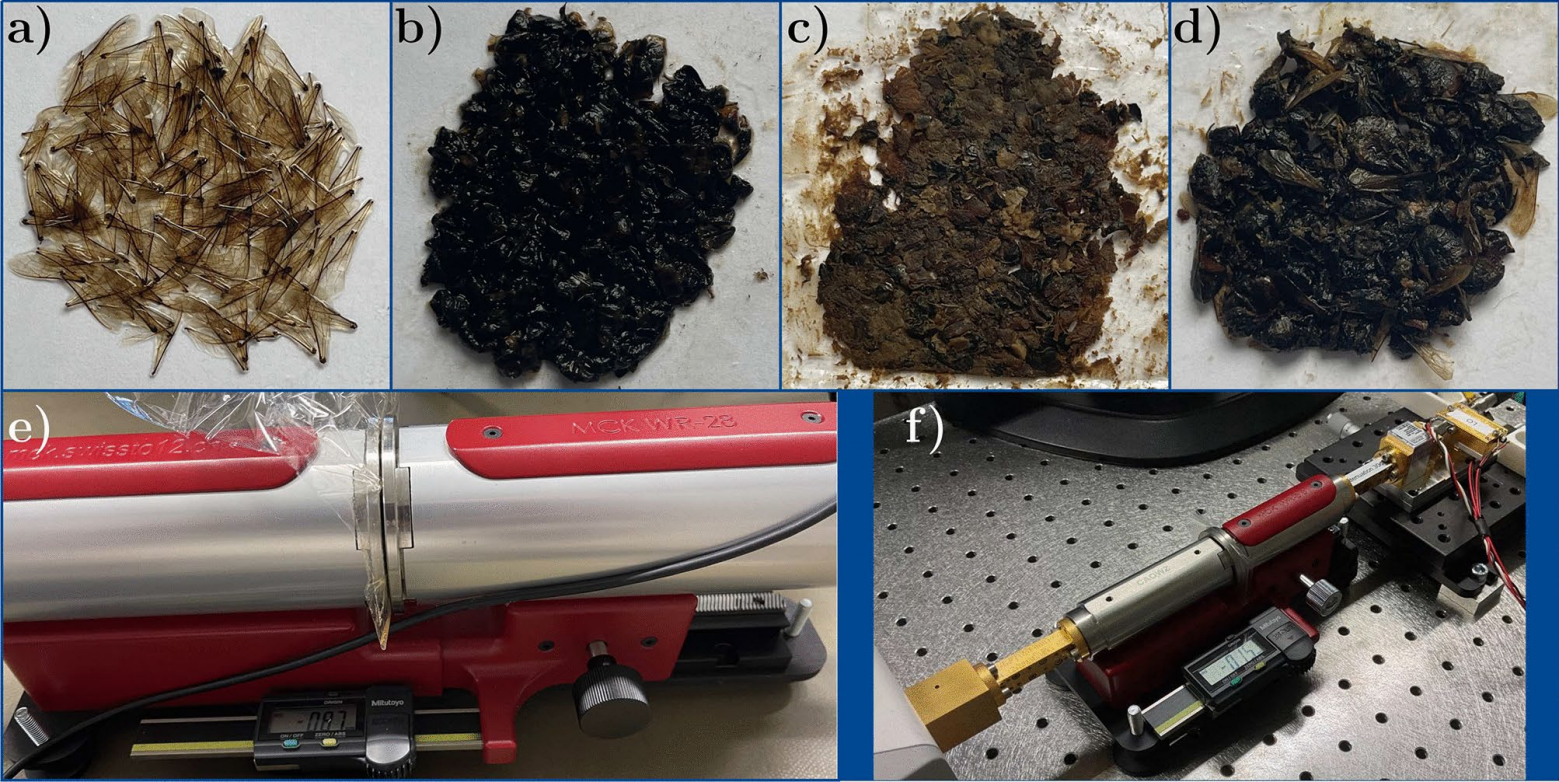
Varieties of bees and their respective parts used as measurement samples: (**a**) wings, (**b**) cuticula, (**c**) inner parts, (**d**) full bee. Displayed in (**e**) is the SWISSto12 MCK system for the K_a_-band, accompanied by the cling foil packaging, while (**f**) showcases the same system for the D-band, featuring the two frequency extenders and accompanied by cling foil packaging.

In light of the rapid expansion in high-frequency wireless communication channels, such as 5G and 6G, reaching up to a few THz, concerns have been raised about the potential impact on arthropods^8,34^, including bees, crucial pollinators in our ecosystem^1,18–20,25,35^. To investigate these interactions and facilitate studies like EM dosimetry^8^ or radar entomology for bee monitoring in ecology and conservation^21^, accurate material properties of insects, particularly European honey bees, at these frequencies are crucial. While limited experimental data exists, covering frequencies up to 40 GHz^21^ and extending recently to 120 GHz^19^, our research has introduced a 5-pole Debye model based on measured data, offering insights into the complex permittivity of aggregated bees across the frequency range of 1–500 GHz [cf.^2^, Section 8]. However, in all the mentioned studies, bees were treated as homogeneous entities, implying that the same material properties were assigned to all bee parts. While this approach has yielded valuable insights into the interaction of European honey bees with high-frequency radiation, particularly in estimating specific EM energy uptake and RCS, critical details such as the effect of the bee exoskeleton (i.e., the cuticula) are currently overlooked. Beyond the first approaches towards EM bee modeling in the mmWave/THz range^2,19,20^, more detailed models - e.g. including the cuticula - are in the verge to get implemented into the digital twins of bees^36^. The oversight of such details may disregard potential shielding effects^36^ when exposed to EM radiation up to THz frequencies, as well as the influence of extremities such as wings, antennae, and legs on the associated EM scattering and absorption cross sections. In this study, a novel approach involves dissecting well-preserved dead bees into their major parts as illustrated in Fig. 2. The test samples were aggregated to form planar and dense flat material probes for wings, cuticula, inner parts and homogenized total bees that are all sealed in ultra-thin cling foil (thickness around 7–10 $\mu$m). The complex permittivity of each part is then measured in the K_a_-band (25–40 GHz) and D-band (110–170 GHz), respectively, using the corresponding material characterization kits (MCKs) from SWISSto12^37^, where the resulting permittivities are to be introduced into heterogeneous bee models for corresponding numerical dosimetric and scattering analyses. In the following individual 5-pole Debye models are fitted to the corresponding measured data sets using evolutionary algorithm-based optimization. This framework allows to extrapolate the permittivities (via the determined individual dielectric functions) to an extended frequency range covering now 1–500 GHz. This effort provides for the first time, a dependable set of reliable, heterogeneous bee material parameters at THz frequencies.

1 $$\begin{aligned} \varepsilon = \varepsilon _\infty + \sum \limits _{i=1}^{5} \frac{\Delta \varepsilon _\textrm{i}}{1+ j \omega \tau _\textrm{i}} + \frac{\sigma _\textrm{s}}{j \omega \varepsilon _\textrm{0}} \end{aligned}$$

Here, $\varepsilon _\infty$ represents the permittivity at an infinite frequency, $\sigma _{\rm s}$ denotes the electrical conductivity, whereas $\varepsilon _{0}$ denotes the permittivity of vacuum, $\omega$ is the angular frequency, $\Delta \varepsilon _{\rm i}$ and $\tau _{\rm i}$ for $i = 1-5$ are the change in the permittivity and relaxation time related to its corresponding relaxation frequency of the Debye dispersion, respectively.

### Development of 3D models

Two distinct 3D models are created for the finite-difference time-domain (FDTD) simulations and RCS measurements, respectively. While a simplified honey bee digital twin may suffice for evaluating RF exposure in various parts of the honey bee’s body and conducting a qualitative analysis of their interaction with EM waves, a highly realistic honey bee model is advantageous for RCS measurements. Such a highly realistic model includes detailed features such as the hairy forelegs, which are essential for pollen removal and collection etc.

**Fig. 3:**
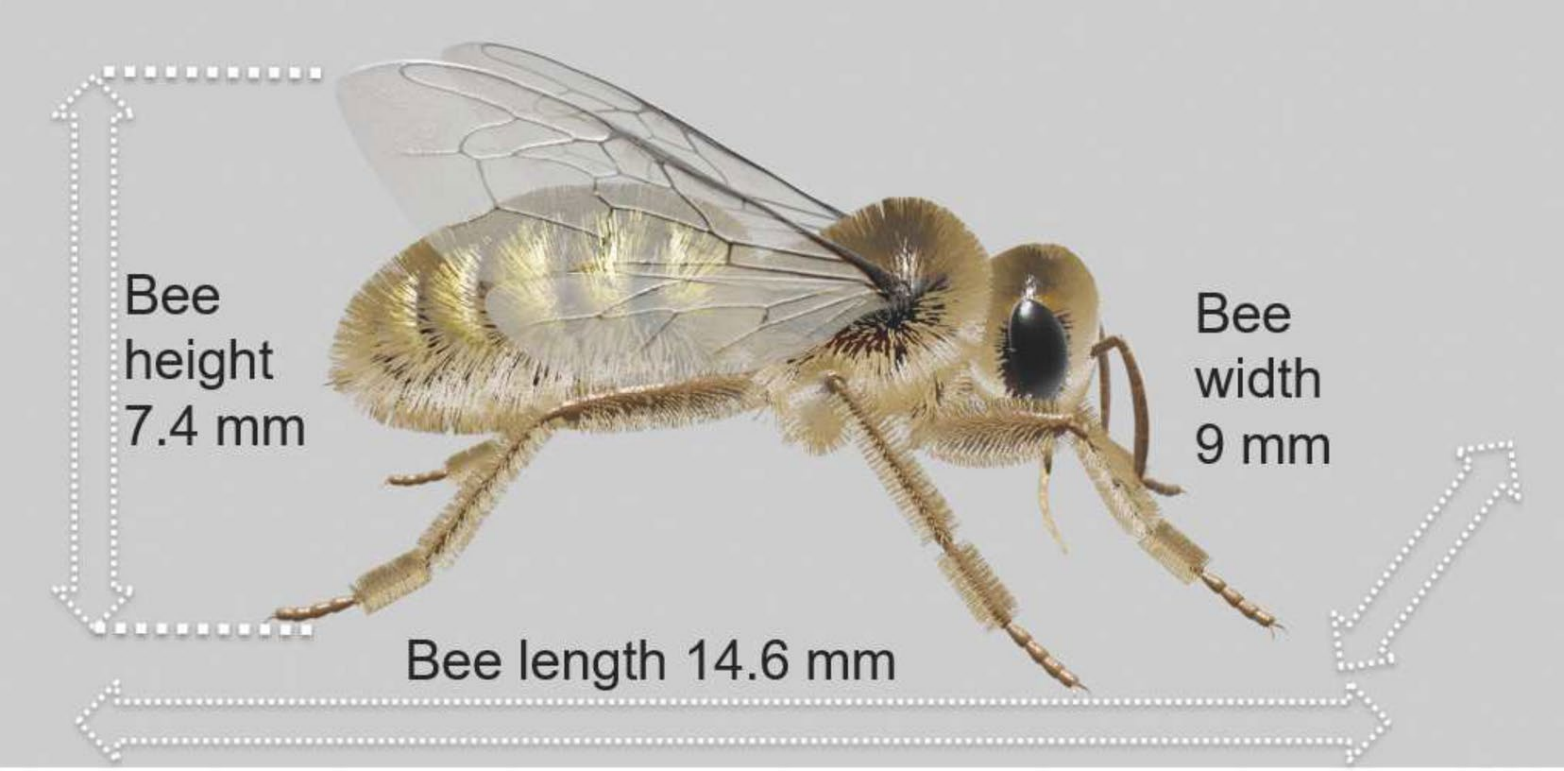
Illustration of the European honey bee digital twin used for AM and RCS measurements.

*Micro-CT honey bee model for FDTD simulations*. The 3D models of European honey bees were created following the procedure detailed in previous work^19,20^. The studied specimen was approximately 11.0 mm long, 5.0 mm wide, and has a mass of 900 mg. A bench-top MicroCT scanner (Quantum GX MicroCT Imaging System, PerkinElmer, Hopkinton, MA, USA) at the Western Sydney University National Imaging Facility (Sydney, Australia) was used to scan the bee. The following parameters were used: 50 kVp, 80 $\mu$A, high resolution 2048 $\times$ 2048 pixels image matrix, with 20 $\mu$m isotropic voxel size. Scanning time was 3 s for each of the 180 projections with 3 s rotation in between each projection. The total scan time was approximately 18 min per whole bee. The Quantum GX, bench-top MicroCT scanner’s software was used to reconstruct the 180 projection images and then convert them into a 2D rendered image stack of 512, 16 bit bitmap images. Bee volume data were then acquired by loading the image stack into BeeView volume rendering software (DISECT Systems Ltd, Suffolk, UK)^19^. Captured images, containing valuable information such as pixels and slice spacing, were imported into the software TomoMask. The transfer from 2D to 3D format was accomplished by applying the arching cubes algorithm^38^, resulting in the export of an STL (STereo Lithography) file commonly used for representing complex surface and volume geometries.

*AM honey bee model for RCS measurements*. The European honey bee model has been meticulously constructed using advanced modeling techniques within the powerful Blender 4.0 (Stichting Blender Foundation, Amsterdam, Netherlands, www.blender.org) software. It embodies increased complexity and a higher degree of realism. This AM honey bee model comprises 57,914 faces and goes beyond being a mere digital replica; it accurately represents the heterogeneous nature of real honey bees with respect to their electrical properties. Such fidelity in modeling renders them invaluable for RCS measurements. The digital twin employed for AM is depicted in Fig. 3.

### Additive manufacturing

**Fig. 4:**
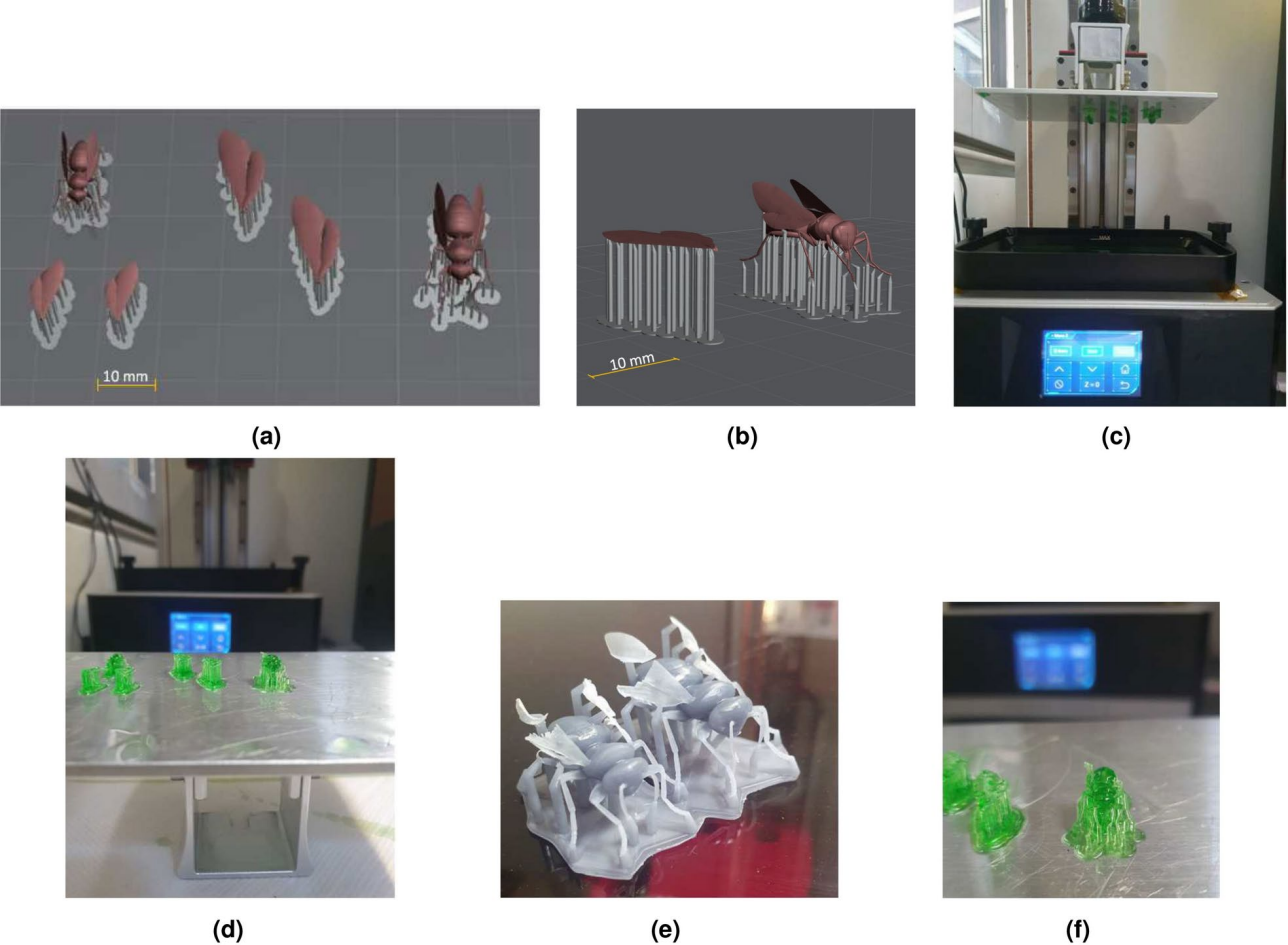
Bee model in virtual preparation space for AM (**a**) topview, (**b**) side view, (**c**) 3D printer with honey bee on corresponding printing platform, (**d**) detached printed honey bee models, (**e**) honey bee models in opaque gray and (**f**) in transparent green resins.

For years, the use cases in AM have been increasing, whether in the automotive industry, aerospace or medical technology. Various processes from AM are used. In the AM process of powder bed-based fusion with plastics (PBF-P), powder is applied to a build platform in a system using a roller or blade. A laser scanner system deflects the laser beam, exposing the contour or surface that belongs to the solid model. After completing each layer, the building platform moves downwards by a defined height, a new layer of powder is then applied, and the appropriate contour or surface is exposed. This process is repeated until the object is completed. The solidified object is finally removed from the powder cake, brushed, and any remaining loose powder particles are removed by blasting them off with glass beads. Material extrusion is a cost-effective process in terms of both purchase and material expenses. In this process, a plastic strand is melted by a heated nozzle and then deposited layer by layer. Another process is, for example, VAT Photopolymerization (VPP), in which a lightcuring resin is solidified in a basin via a light source such as a laser or ultraviolet (UV) light. For these and other AM processes that build the component layer by layer, an initial virtual object file is utilized. Creating a 3D object involves using a design program, followed by breaking it down into individual layers using a suitable software. The slicer software, as it’s commonly referred to, allows for setting parameters such as layer thickness, speeds, temperatures, etc., depending on the specific process^39,40^. Due to the small size of the bees, VPP is employed for producing the bee models. In the slicer software, a layer thickness of 25 $\mu$m is chosen for both the bees and the separately produced wings. Figure 4a illustrates a section of the virtual build space with two different bee sizes and their individual wings.

The support structures, shown in light gray, are detailed in the enlargement in Fig. 4. Since the wings have a thickness of 25 $\mu$m, they are manufactured individually to achieve optimum alignment for production. In the AM process, holes and similar geometries are manufactured along the build direction with the highest quality. For the bee, an upside-down alignment would be most optimal. However, a trade-off is made between the surface to be examined and the use of support structures. Components manufactured using VPP typically require support structures. The bee is positioned, as depicted in Fig. 4b, to ensure that these structures do not affect the top surface of the bee. In Fig. 4c, the “3D printer” is visible without the hood, protecting the material from UV light radiation. The build platform in the upper area depicts objects made of green resin. Figure 4d illustrates the detached build platform, featuring the bee and wing models, emphasizing that the wings are positioned with support structures on their underside as flat as the geometry allows on the building platform. For the subsequent experiments, the bee model is created in its original size and magnified 1.5 to 2.5 times. In the studies involving the bee models, two resin colors are used for fabrication: transparent green and opaque gray (refer to Fig. 4e,f). In the opaque gray bee model, it is evident that the wings cannot be manufactured with the set layer thickness in the resulting orientation. Hence, a two-part model was chosen allowing the wings to be glued on afterward. In addition to the resin bee models, these are also manufactured from the standard material PA12 using the PBF-P process, characterized by its very good mechanical properties, processing properties and heat resistance^40,41^. During manufacturing, the bees are positioned with their upper body surface facing downwards, resulting in improved surface qualities on the contours and surfaces facing downward. These bees are manufactured with a set layer thickness of 0.1 mm. This results in a roughness $R_{\rm z}$ of approx. 74 $\mu$m in the z-direction and approx. 77 $\mu$m measured on the top side in the x-y-direction. Those roughness results correspond to the state-of-the-art^42,43^. Due to the geometry and size of the bee models, it was not possible to carry out roughness measurements on them.

### RCS measurements

*THz-TDS measurements*. The THz band of the EM spectrum is very attractive for radar applications due to its broad bandwidth and shorter wavelength. These features enable finer range and cross-range resolution, facilitating more precise detection and classification of small objects, such as insects. Prior research in the mmWave frequency range (12 GHz, 24 GHz, 77 GHz)^24–27^ has explored the RCS to monitor the movement of large insects where the wavelength corresponds to the size of the insects. As a result, detecting finer details of the insects becomes challenging. Therefore, it is necessary to shift to higher frequencies, such as the THz band, to accurately detect and classify smaller insects. In previous experiments, measurements were carried out on a deceased bee to determine its RCS. However, the bee’s material properties and shape possibly changed during the process as it was immersed in ethanol to prevent desiccation^2^. While being measured, the ethanol evaporated, causing the bee to undergo drying. For the current study, 3D-printed honey bees crafted from UV resin and PA12 are employed as substitutes for real honey bees. These honey bee models ensure consistent measurement results by maintaining reliability over time. The production using AM ensures stable shaping and dielectric properties of the samples. Statistical analysis can reveal deviations between samples and evaluate the precision of AM production. In addition to introducing a heterogeneous honey bee model based on non-homogeneous dielectric properties, this work primarily focuses, from a measurement perspective, on the interaction of honey bee models with THz waves, with particular emphasis on aspects such as RCS, imaging, and spectral properties as distinguishing features for reliable honey bee monitoring. Noteworthily, the RCS characteristics are dominated by the feature size or structure of the target rather than the material properties themselves. Therefore, for example, broadband THz-TDS measurements with mock-ups are necessary to estimate the required frequency band for later radar-based methods. The initial phase involves conducting simple reflection measurements to characterize the body of the honey bee. The all-fiber-coupled THz-TDS system, TeraK15 from Menlo Systems, is utilized for the generation and detection of THz radiation. A mode-locked fiber laser generates sub-100 fs laser pulses at a repetition rate of 100 MHz. The laser pulse is then converted into the THz frequency range by a photoconductive switch serving as the transmitter (Tx). For detection, a second photoconductive switch functions as a receiver (Rx) alongside a delay line, enabling temporal sampling of the THz pulse. The incident field, proportional to the measured photocurrent, allows the generation and recording of an EM spectrum ranging from 100 GHz to 6 THz. A detailed overview is given by Balzer et al.^28^.

**Fig. 5:**
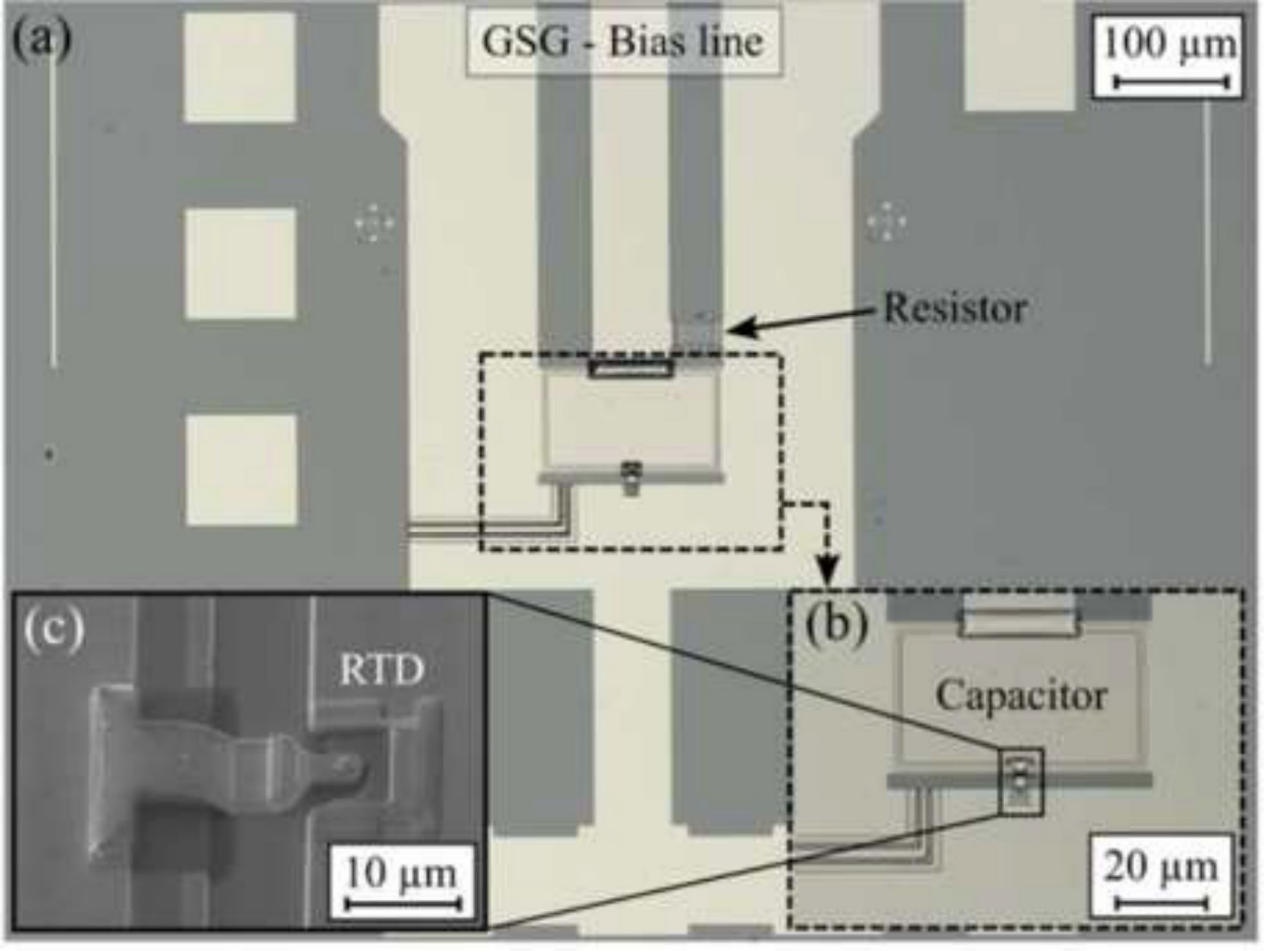
Optical microscope image of the RTD oscillator chip (**a**), close-up view of the slot antenna (**b**), and electron microscope image (**c**) of the RTD integrated into the slot antenna. $\copyright$ [2023] IEEE. Reprinted, with permission, from^53^.

*Frequency domain measurements with RTD THz source*. Practical applications of THz technology in open environments demand compact designs for easy integration and portability while also emphasizing power efficiency. Electronic THz oscillators show significant potential for applications such as advanced communication, radar systems, and imaging, offering a wide frequency tuning range, high output power, efficiency, and compact design^44^. Diverse technologies, including RF CMOS, SiGe HBT, and InP HBT or HEMT transistor technologies have been investigated for THz oscillators, allowing for frequencies beyond 1 THz in M-push oscillator designs^45^. There is always a trade-off between output power and oscillation frequency, limiting efficiency with higher multiplication factors beyond the transistor’s maximum frequency of oscillation ($f_{\rm max}$). Beyond $f_{\rm max}$, a promising alternative is provided by RTDs, where the transit time is constrained by the device’s quantum capacitance rather than electron transport through a depleted region, as in transistors. Currently, RTDs stand as the highest-frequency solid state electronic oscillator, achieving nearly a 2 THz self-oscillation frequency, even at room temperature, as demonstrated in multiple research studies^46–49^. Other published works have shown a fundamental oscillation at 1.55 THz with optimization of the antenna length, yielding an output power of $\thicksim$600 $\mu$W at 620 GHz with a two-element offset-fed antenna array. They also predicted RF output power exceeding 1 mW at 600 GHz for an oscillator combining an RTD with a slot antenna^50,51^. Recently, power combining techniques have allowed emitted power over 10 mW at 450 GHz with a DC-to-RF conversion efficiency of 1$\%$^52^. The current-voltage (IV) curve of the RTD exhibits a region of negative differential resistance (NDR). When biased in this region, the NDR offsets the load of the antenna, and the diode forms a self-oscillating resonator between the diode’s junction capacitance and the inductance of a chip-integrated planar antenna. On-chip antenna integration results in an ultra-compact oscillator, exhibiting high power efficiency, as the oscillator operates at its fundamental frequency. For this study, in-house fabrication of InP RTD chips with integrated slot antennas was performed. Double barrier RTD layer stacks were grown on semi-insulating Fe-doped InP (100) substrates using molecular beam epitaxy. The layer stack includes a sub-well system of InGaAs/InAs and AlAs barriers with a nominal thickness of 1.4 nm, as illustrated in Fig. 5. Top-down processing was employed to create the RTD diode and the antenna using standard optical lithography and wet chemical etching. Other details of the fabrication process were published in previous work^53^.

## Results

**Fig. 6:**
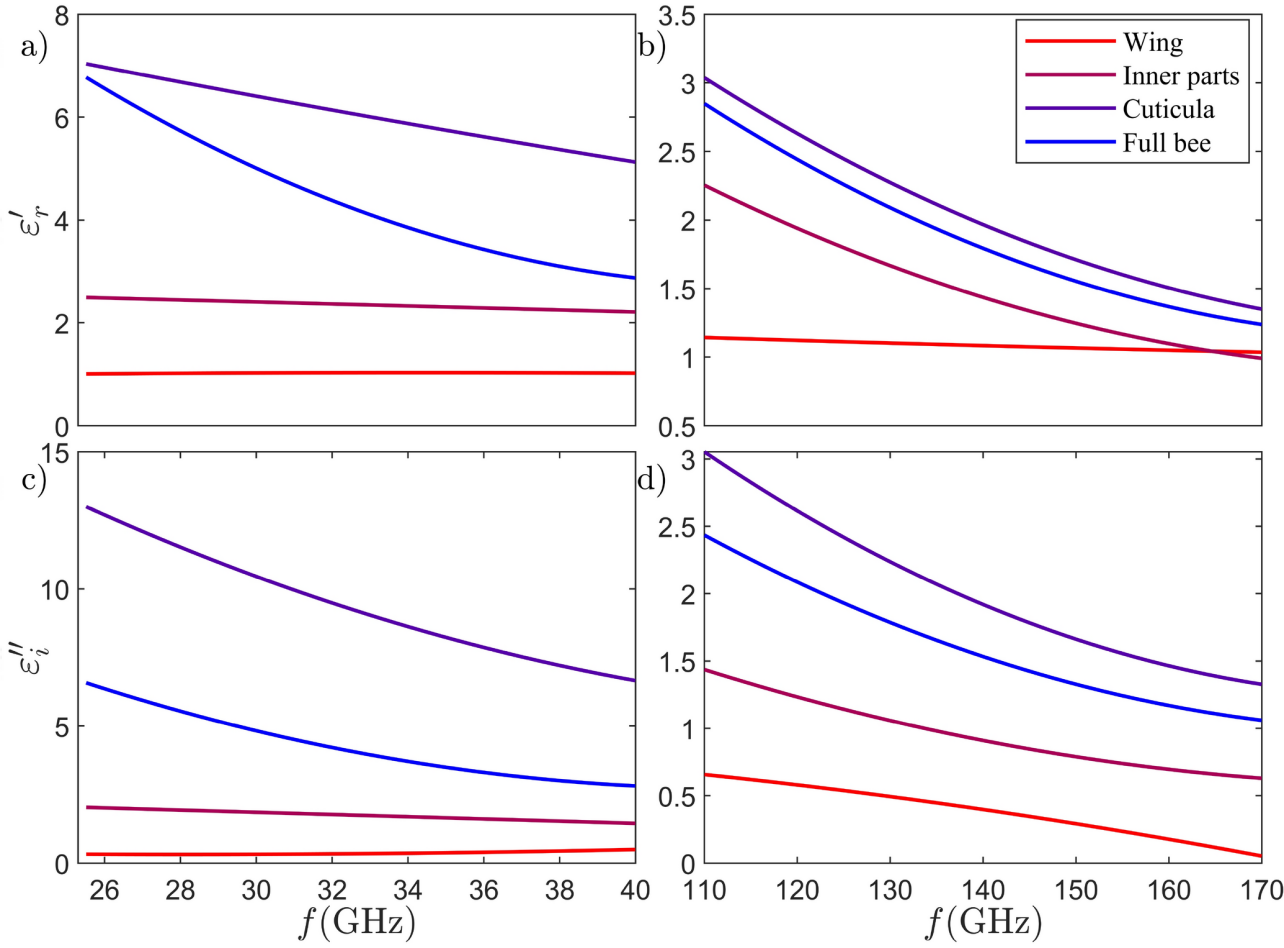
The determined complex permittivities for various body parts of European honey bees, showcasing the real part in (**a**) the K$_{\rm a}$-band and (**b**) the D-band, alongside the imaginary part depicted in (**c**) the K$_\textrm{a}$-band, and (**d**) in the D-band.

### Dielectric properties

The SWISSto12 MCKs comprise two opposing corrugated circular waveguide horn antennas that support a HE$_{11}$ mode, characterized by low propagation loss and a uniform wave front at the measurement gap between the horns^54,55^. There is a small concern to be considered here, as the measurement gap in the MCK setup gives rise to a lateral power leakage, which leads to a slight but systematic overestimation of the probe sample’s loss tangent (resp. $\varepsilon ''$). This has neither been addressed by the manufacturer SWISSto12 nor by the many users of this system. In a separate study we have verified the existence of this parasitic radiation^56^ by corresponding near- and far-field measurements and concluded that the power leakage becomes increasingly negligible for decreasing gap sizes, which fits well to our thin probe scenarios. Connected to the Rohde & Schwarz vector network analyzer (VNA) ZVA40, the MCK incorporates two frequency extender modules for the D-band—namely, the ZC170 in the transmitting path and the ZRX170L module from RPG—in the receiving path (cf. Fig. 2f). The material under test (MUT) is precisely fitted into the gap between the waveguide horns, with the gap size (i.e., MUT sample thickness) determined by the built-in precision caliper with a 10 $\mu$m accuracy. *S*-parameters for each sample are recorded in both frequency bands following two calibration measurements (open and shortened horn, the latter involving a metallic sample). Freshly deceased worker honey bees are sourced from local beekeepers and preserved in a 70$\%$ ethanol solution. These bees are then dissected into major parts such as wings, the exoskeleton (cuticula), and inner parts. Additionally, some bees are kept whole and measured as an ensemble.

As shown in Fig. 2, all samples are sandwiched between circular cling foil packaging conforming to the 4 cm antenna opening of the larger MCK system (K_a_-band). The samples are left outside the ethanol solution for about 12 hours before measurements allowing the ethanol to evaporate without drying the samples. The recorded S-parameters, specifically *S*$_{11}$ and *S*$_{21}$, undergo time gating after calibration to eliminate undesired reflections related to MCK fixtures and end connectors. The complex permittivity of each sample is subsequently determined based on the dielectric measurement method developed by the National Institute of Standards and Technology (NIST), utilizing both *S*$_{11}$ (*S*$_{22}$) and *S*$_{21}$ (*S*$_{12}$) data^54,57^. The calculated complex permittivities for all four samples are presented in Fig. 6. In order to extrapolate the determined complex permittivity across the entire frequency range of 1–500 GHz, a 5-pole Debye model is obtained for each sample through an evolutionary algorithm-based optimization process^58^. The fitness function *F* to be maximized is defined as follows:

2 $$\begin{aligned} F = \left( \sum \limits _{i=1}^{N_\textrm{num}} \frac{1}{N_\textrm{num}} \left( \frac{|\varepsilon _\textrm{mes}'-\varepsilon _\textrm{deb}'|^2}{\varepsilon _\textrm{mes}'^2} + \frac{|\varepsilon _\textrm{mes}''-\varepsilon _\textrm{deb}''|^2}{\varepsilon _\textrm{mes}''^2}\right) \right) ^{-1} \end{aligned}$$

**Fig. 7:**
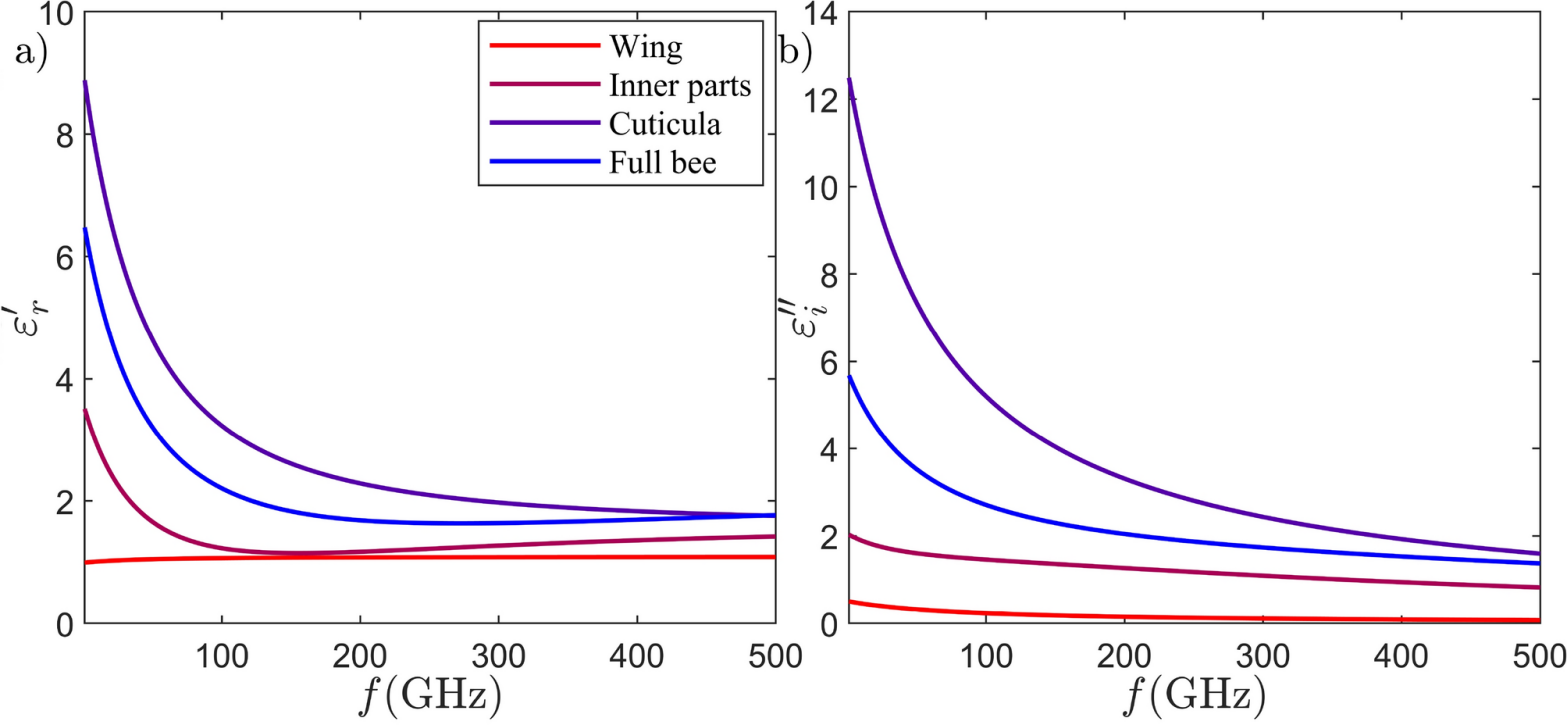
The retrieved complex permittivity of European honey bees: (**a**) real part, and (**b**) imaginary part.

**Table 1:** The coefficients of the fitted 5-pole Debye model for the first five terms in the equation 1.

Bee parts	$\varvec{\varepsilon _\infty }$	$\varvec{\Delta \varepsilon _\textrm{1}}$	$\varvec{\Delta \varepsilon _{2}}$	$\varvec{\Delta \varepsilon _{3}}$	$\varvec{\Delta \varepsilon _{4}}$	$\varvec{\Delta \varepsilon _{5}}$	$\varvec{\tau _{1}}$ (ps)	$\varvec{\tau _{2}}$ (ps)	$\varvec{\tau _{3}}$ (ps)	$\varvec{\tau _{4}}$ (ns)	$\varvec{\tau _{5}}$ ($\mu$s)	$\varvec{\sigma _{s}}$ (S/m)
**Wing**	9.65	956	976	828.5	94.5	790.5	625	999.5	241.5	66.5	340.5	15.6
**Inner parts**	1.61	452	737.5	899	694.5	983.5	3.5	63	266.5	299.5	761	15.6
**Cuticula**	1.59	373.5	10	70.5	288.5	773	416	232	894.5	79.5	698.5	15.6
**Full bee**	2.19	265.5	9.5	608	975.5	918.5	42	16.5	531.5	555.5	187	13.6

Here $N_{\rm num}$ is the number of frequency points, $\varepsilon _{\rm mes}'$ denotes the measured real part of the permittivity, $\varepsilon _{\rm deb}'$ is the corresponding value of the Debye model, $\varepsilon _{\rm mes}''$ represents the measured imaginary part of the permittivity, and the $\varepsilon _{\rm deb}''$ refers again to the Debye model. The complex dielectric functions represented by the fitted 5-pole Debye models for the wings, inner parts, cuticula, and the whole bee are illustrated in the Fig. 7 the frequency range of 1–500 GHz. The corresponding coefficients of the four fitted Debye models are provided in the Table 1. Upon scrutinizing the results presented in Fig. 7, it becomes evident that the cuticula exhibits the highest real part of permittivity among the measured bee parts. This aligns with its firmer chitin structure and specific composition, namely i.e. sclerotized proteins in the chitin layer, compared to the rest of the bee parts. The cuticula also demonstrates the highest imaginary part of permittivity, whereas the inner parts exhibit comparatively lower complex relative permittivity. The extent to which this leads to EM shielding at THz frequencies is currently under intensive investigation (see the next sections). The wings exhibit a low and even comparable complex permittivity to air, suggesting a weak contribution to the EM scattering cross-section. These variations in the complex relative permittivity of each bee part highlight European honey bees as heterogeneous entities from an EM perspective. They consist of sections with significantly different complex relative permittivities, emphasizing the importance of incorporating this heterogeneity into future models for microdosimetric assessment. A key novelty of this work lies in demonstrating that honey bees are indeed heterogeneous entities. The thorough and extensive material characterization proves vital for predicting EM-induced stress on honey bees in the THz region. Consequently, this material characterization enhances the accuracy of full-wave computational EM simulation.

### 3D models

**Fig. 8:**
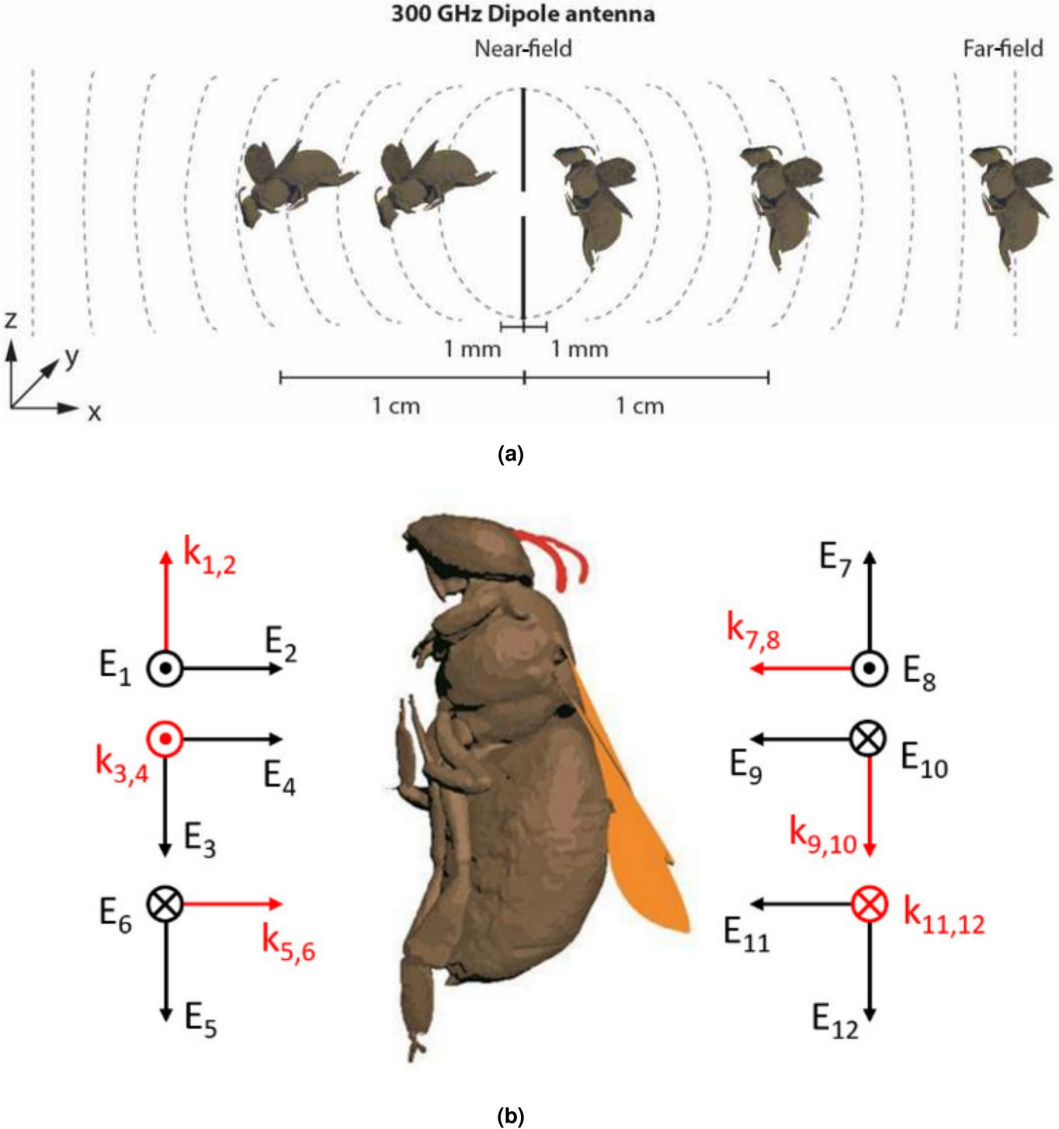
(**a**) Configuration of the FDTD simulations. Honey bee and antenna dimensions are not drawn to scale. (**b**) Configuration of the twelve plane waves in far-field FDTD simulations for the 3D model with closed wings (orange). The plane-wave direction of propagation is indicated by red arrows, while the electric field polarization is indicated with black arrows.

THz-monitoring of insects is an emerging technology. This application involves exposing insects to the fields emitted by THz sensors. To obtain an initial estimate of this exposure, a series of numerical simulations were conducted, simulating a European honey bee worker in the proximity of an antenna emitting at 300 GHz. The honey bee 3D model is based on the homogeneous, single-tissue model developed in^20^, obtained using micro-computerized tomography. The current model is an adaptation of this previous version, incorporating four anatomical components: wings, antennae, exoskeleton, and inner tissues. The dielectric properties of the model are listed in Table 2. The dielectric properties of the exoskeleton were assigned to the antennae, outer part of the legs, and body, while the inner part was characterized by inner tissue and the wings by wing tissue. The exoskeleton thickness was set at 0.05 mm, striking a balance between realism^59^ and feasible simulation time. Two versions of the bee model are employed in this work: one with open wings (see Fig. 8a) and one with closed wings (see Fig. 8b), emulating wing movement in flight. Since the wings were not accurately reconstructed from the micro-CT, as detailed in^20^, they were manually added using two different methods. For the open-wing model (Fig. 8a), the wings were extracted from another honey bee model in the Sketchfab database. In the closed-wing model (Fig. 8b), the wings were based on anatomical pictures and modeled using Blender and Autodesk Netfabb Premium (Autodesk, San Francisco, USA, www.autodesk.com.) These wings had lower mesh density and were smoother compared to the wings used in the open-wing model. They were placed in a closed position, as seen in Fig. 8b.

**Table 2:** Dielectric properties used in the numerical simulations at 300 GHz.

Bee parts	$\varvec{\varepsilon _\textrm{r}}$	$\sigma _\textrm{s}$ (S/m)
Exoskeleton	1.973	40.61
Wings	1.080	1.752
Inner tissues	1.637	28.94

### Numerical simulations

**Fig. 9:**
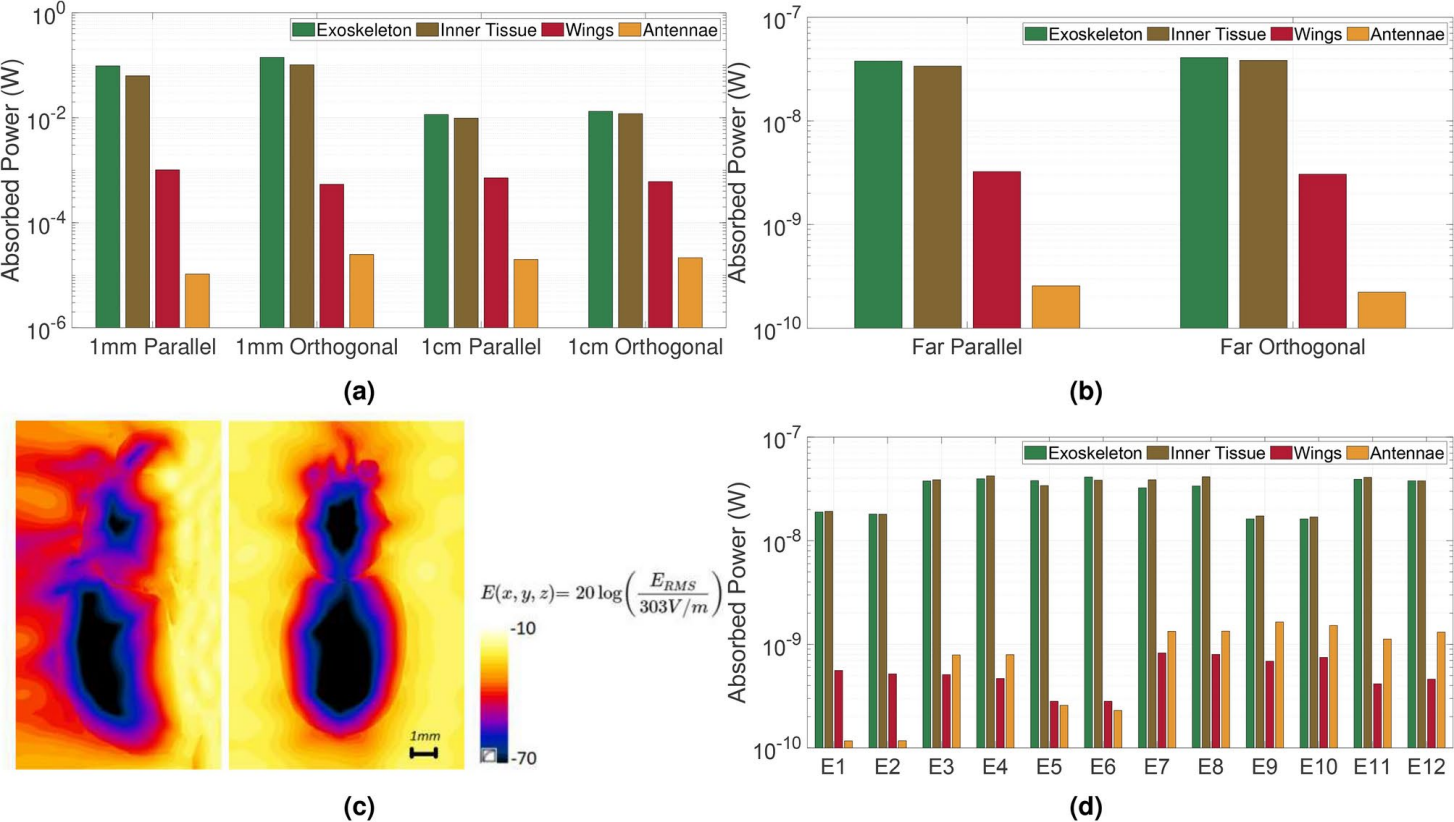
Absorbed power in four different tissues at 300 GHz for (**a**) four near-field configurations with 1 W input power, (**b**) corresponding far-field polarizations with an incident electric field strength of 1 V/m, (**c**) mid-sagittal (left) and mid-coronal (right) cross-sections of the electric field distribution at 1 cm separation distance from an orthogonally polarized dipole antenna that is fed an input power of 1 W at 300 GHz. (**d**) Absorbed power in different tissues of the honey bee (with closed wings) for all considered far-field polarizations with an incident field strength of 1 V/m at 300 GHz.

FDTD simulations were carried out in Sim4Life 7.2.1.11125 (ZMT, Zürich, Switzerland, https://sim4life.swiss/) to calculate the radio-frequency EM fields (RF-EMFs) inside the heterogeneous honey bee model and assess the EM power absorbed ($P_{\rm abs}$) by the insect at a frequency of 300 GHz. The simulations were split into two categories: near-and far-field simulations. In the near-field simulations, the open-wing honey bee model was positioned at distances of 1 mm and 1 cm from a dipole antenna, measured from the ventral side of the bee. For each separation distance, two orthogonal polarizations of the antennas were considered, as depicted in Fig. 8a. As the RF-EMF source, a half-wavelength dipole antenna designed to operate at 300 GHz with a power reflection coefficient $|S _{11}|^2$ lower than -15 dB was used. The antenna had a dipole arm length of 0.21 mm, an arm diameter of 0.01 mm, and a feed diameter of 0.001 mm. The simulation duration ensured that all simulations reached a steady-state solution. To achieve this, the electric field along a line positioned 0.8 mm behind the bee was monitored, resulting in 24 simulation periods at 1 mm and 42 periods at 1 cm. For both separation distances, the grid step was set at 0.05 mm, well below the stability criterion, and measures were taken to ensure proper connection of the exoskeleton. The absorbed power averaged across four tissues is presented in Fig. 9 for all near-field configurations and, in the far-field, for the corresponding two polarizations. As expected, in accordance with previous findings^60^, the absorbed power diminishes with increasing distance.

**Table 3:** Absorbed power and relative absorbed power in the near-field for different tissues at an input power of 1 W through the dipole antenna for the open wing model and the averaged $P_{\rm abs}$ for the 12 plane waves and relative absorbed power for different tissues with an electric strength of 1 V/m in the far-field for the closed wing model.

Bee parts	Volume (mm$^3$)	Distance 1 mm PP		Distance 1 mm OP		Distance 1 mm PP		Distance 1 mm OP		Far field All Polarizations	
		AP (W)	RAP (%)	AP (W)	RAP (%)	AP (W)	RAP (%)	AP (W)	RAP (%)	AP (W)	RAP (%)
BA	0.08	1.05E-5	0.0065	2.49E-5	0.0102	2.00E-5	0.0905	2.15E-5	0.08	8.81E-10	1.37
ES	6.29	0.0974	60.3	0.141	57.9	0.0115	52.2	0.00132	51.2	3.07E-8	47.9
IT	47.9	0.0631	39.1	0.102	41.9	9.83E-3	44.5	0.00120	46.3	3.20E-8	49.8
WI	Open: 1.92 Closed: 0.45	1.01E-3	0.63	5.43E-4	2.22	7.17E-4	3.24	6.09E-4	2.3	5.46E-10	0.85
Total		0.161		0.244		0.220		0.0258		6.41E-8	

Abbrevations: BA = Bee Antennae, ES = Exoskeleton, IT = Inner Tissue, WI = Wings, PP = Parallel Polarization, OP = Orthogonal Polarization, AP = Absorbed Power, RAP = Relative Absorbed Power

At 300 GHz, the penetration depth significantly limits the absorbed power in the inner tissue as confirmed by the sagittal and coronal cross-section in Fig. 9. Particularly, in the far-field, the inner tissue absorbs very little relative to its volume, as indicated in Table 3. The wings exhibit minimal absorption due to their small volume and low conductivity. Nevertheless, in the far-field wing absorption increases, as waves are more likely to impinge directly on them than when shielded from the source and positioned farther away, as is the case in the near-field. At 300 GHz, there is minimal difference between the two polarizations. However, at lower frequencies, particularly around the resonance frequencies of the bee (i.e. when the wavelength is equal to the bee’s dimensions), there tends to be a significant difference between these polarizations^61^. In the near-field, the exoskeleton and inner tissues absorb the majority of the power, with the power distribution being almost even between them. The far-field of an EM source is located at a distance greater than the Fraunhofer limit, which is approximately 738 mm here. For far-field exposure, a set of incident plane waves can represent the EM exposure. Following the strategy used in^20^, twelve plane waves were selected for this purpose, as depicted in Fig. 8b. This set of plane waves includes incident angles and polarizations along the main axis of the bee, aiming to capture the minimum and maximum possible $P_{\rm abs}$. Due to simulation time constraints, we opted to work with the 3D model with closed wings to reduce the simulated space. The extra simulations included the open wings model, considering a single incident angle and two polarizations. These conditions were chosen to align with the far-field depicted in Fig. 8, in particular, with plane waves 5 and 6 from Fig. 8b. In FDTD simulations, a voxel size of 0.05 mm was applied to the body and open wings, and 0.025 mm to the closed wings, ensuring seamless connectivity without disruptions. The simulation ran for 25 periods to reach a steady state, with the root mean square (RMS) of the incident electric field strength set at 1 V/m.

**Fig. 10:**
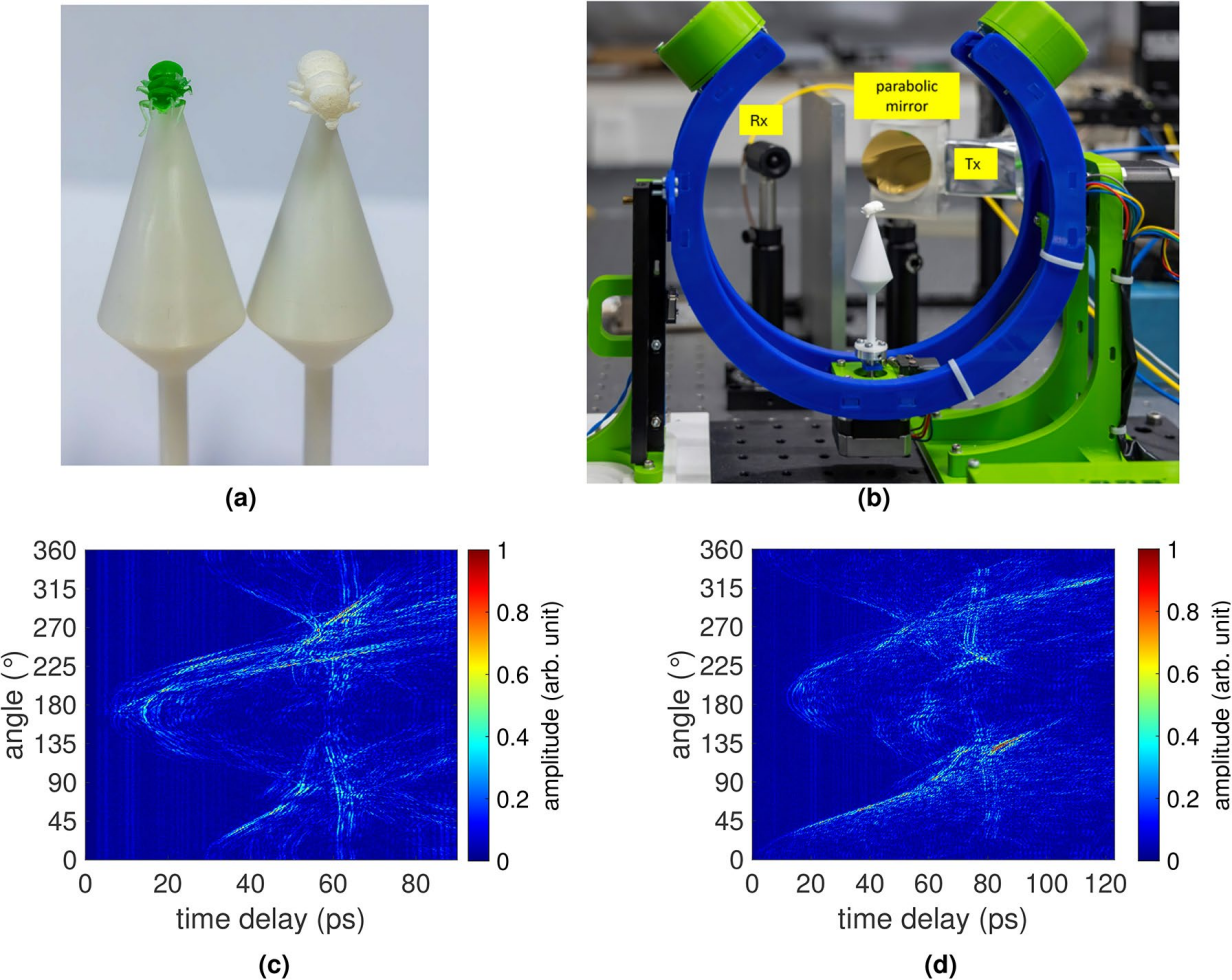
Measurement setup featuring (**a**) a photo of honey bee models constructed with resin (green) and PA12 (white) materials mounted on the holder, and (**b**) side-view of the spherical THz-TDS system. Frequency-gated radargrams depicting the (**c**) green UV resin and (**d**) white PA12 honey bee models, derived from the averaged traces at 0° elevation for all azimuth positions.

The far-field simulations yielded a whole-body $P_{\rm abs}$ of 6.41 × $10^{-8}$ W, averaged over the 12 plane waves. The closed-wing bee, exposed to incident fields of 1 V/m, exhibited minimum and maximum values of 3.54 × $10^{-8}$ W and 8.15 × $10^{-8}$ W respectively. At 300 GHz, the penetration depth limits absorbed power in the inner tissue, constituting 85% of the bee’s volume but absorbing only 50% of the total power (as indicated in Table 3). The wings, with their small volume and low conductivity, contribute minimally to absorbed power. Comparisons with whole-body $P_{\rm abs}$ results in^20^, conducted at frequencies between 2 GHz and 120 GHz, show slightly smaller values at 300 GHz, aligning with the trend observed in^20^. This validates the dielectric properties’ choice in^20^. In^60^, simulations of a honey bee near a dipole antenna at frequencies 6–240 GHz indicated an absorbed power of 0.025 W at 240 GHz and a separation distance of 10 wavelengths. This aligns well with our study’s values at a 1 cm separation distance. Figure 9d illustrates volume-averaged absorbed power values in honey bee tissues for all considered EF polarizations from Fig. 8b. The ICNIRP reference levels for the general population set a 10 W/m^2^ incident power density at 300 GHz, equivalent to a RMS electric field (RMS EF) strength of 61.4 V/m for far-field plane waves. Adjusting the absorbed powers calculated in the far-field simulations to this electric field strength yields a total absorbed power of 0.29 mW in the parallel polarization and 0.31 mW in the orthogonal polarization in the honey bee. In the near-field, an antenna with input power of 1.8 mW and 1.3 mW for the parallel and orthogonal polarizations, respectively, at a 1 mm separation distance, results in an equal absorbed dose. At a 1 cm separation distance, the corresponding values are 13.2 mW and 12 mW for the parallel and orthogonal polarizations, respectively.

### RCS measurements

*THz-TDS measurements*. For RCS measurements, Fig. 10 illustrates the use of a spherical inverse synthetic aperture to rotate the honey bee model in azimuth and elevation planes within the measurement setup’s constraints^62^. This facilitates an angle-dependent broadband RCS measurement while ensuring the honey bee remains at the center of rotation. The antennas are arranged in a bistatic configuration with a 100 mm separation between the Tx and Rx. Additionally, both the collimated beam and the Rx direction are aligned towards the center of rotation, resulting in a 28° angle between Tx and Rx. The antennas are positioned 200 mm away from the center of rotation. A parabolic mirror with a focal length of 50.8 mm collimates the divergent beam from Tx. The honey bee model, affixed to a sample holder, is situated within this collimated radiation. To minimize the reflections from the sample holder towards the Rx, a cone structure with a small tip as shown in Fig. 10b, is employed. The Anycubic white ABS resin and a VPP printer were employed for fabrication through AM. Due to the resin printing, the surface is very smooth, ensuring no scattering occurs at the sample holder and only the object under test (OUT) scatters the incoming radiation. In the measurement setup shown in Fig. 10b, the honey bee models (UV resin and PA12) are rotated by a step motor in azimuth by 360° in 0.5° steps, maintaining a fixed elevation at 0°

At each azimuthal position, 6000 THz-TDS traces are recorded and averaged. In Fig. 10, the recorded THz-TDS traces for all measured azimuth angles, also called radargrams, are depicted to visualize possible reflections from the honey bee. Figure 10c shows the radargram of the green UV resin honey bee, whereas Fig. 10d shows the radargram for the PA12 honey bee. For both models, the reflections at the sample holder are negligible in the radargram. Furthermore, for every angle step, a reflection from the honey bee can be seen. Nevertheless, the intensity of the reflection varies with the angle, suggesting that the bees do not exhibit consistent reflectivity in each direction. To explore this behavior, the RCS will be calculated for different frequencies. To mitigate reflections from the sample holder, a windowing mitigate process is applied to the presented radargram. This enhances the accuracy of the calculated RCS and ensures precise computations.

**Fig. 11:**
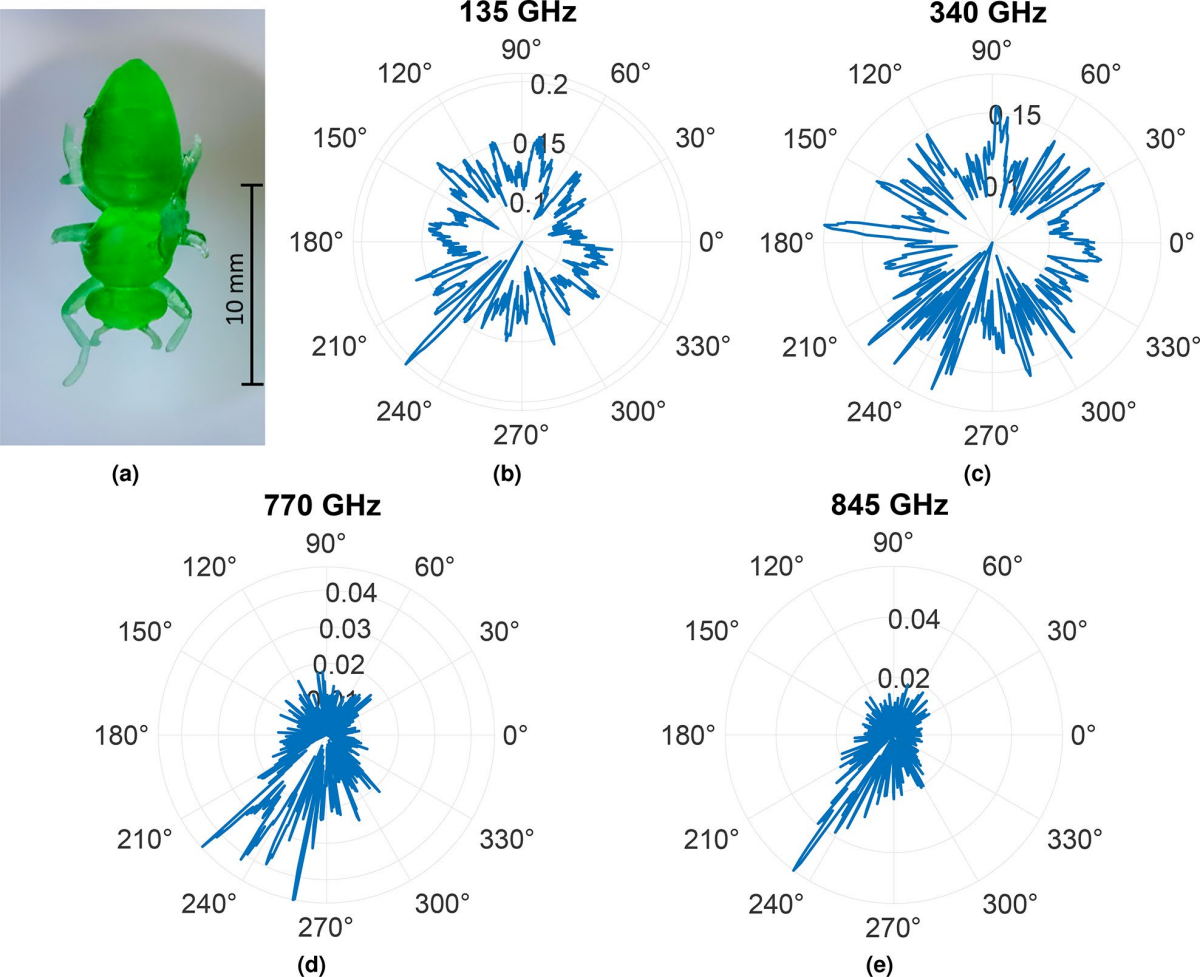
Measured bistatic RCS for (**a**) UV resin honey bee model as a function of the azimuth angle at frequencies (**b**) 135 GHz, (**c**) 340 GHz, (**d**) 770 GHz, and (**e**) 845 GHz.

**Fig. 12:**
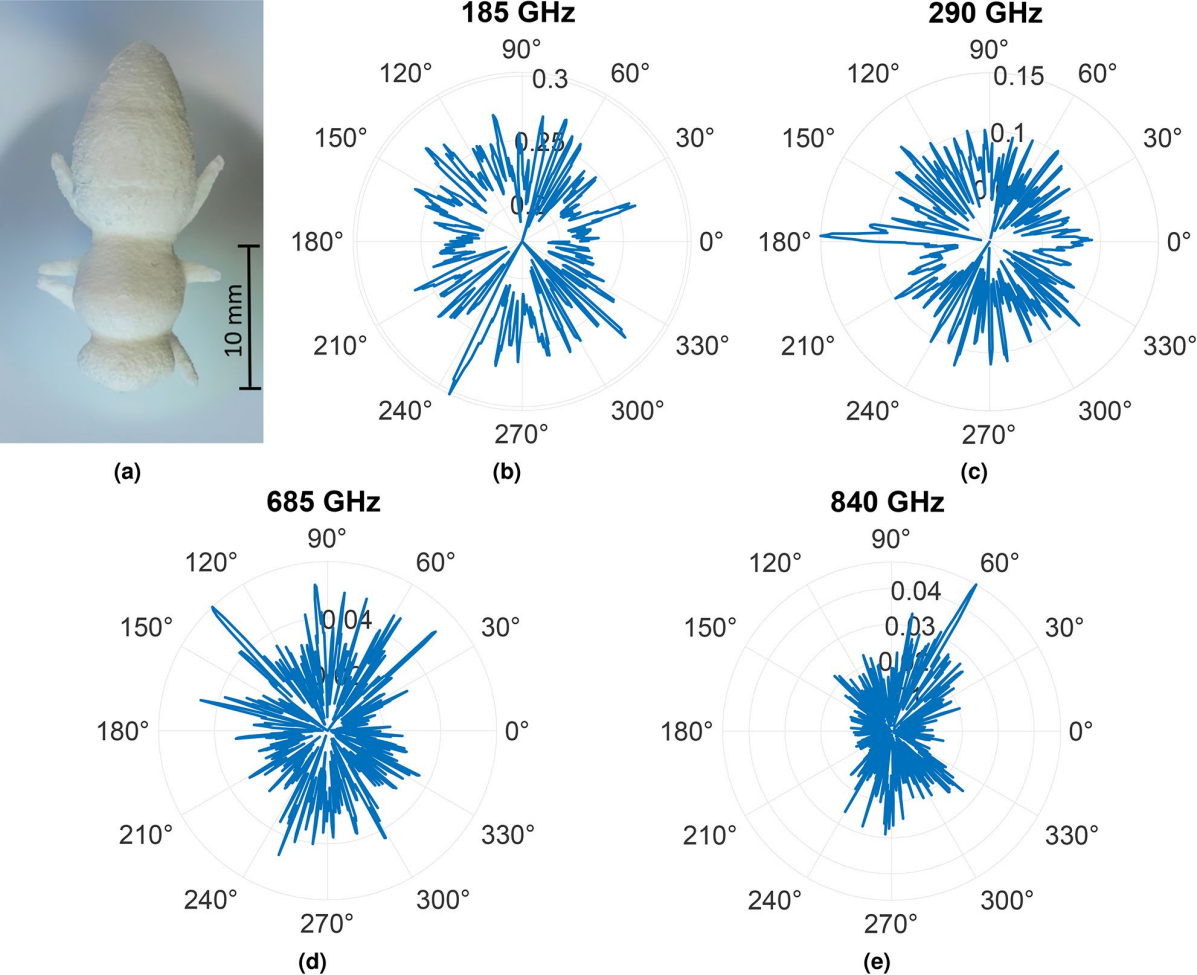
Measured bistatic RCS for (**a**) PA12 honey bee model as a function of the azimuth angle at frequencies (**b**) 185 GHz, (**c**) 290 GHz, (**d**) 685 GHz, and (**e**) 840 GHz.

Figures 11 and 12 represent RCS equivalents for the respective UV resin (green) and PA12 (white) honey bee models at different frequencies where the reflected intensity is normalized by the reference measurement conducted against a metal plate. Here, the measurements are carried out with the honey bee’s abdomen aligned in the 0° direction. Upon comparative analysis, in the case of the 135 GHz frequency, the green UV resin honey bee exhibits high reflectivity from its legs at 227°, whereas the white PA12 honey bee shows high reflectivity from the legs at 245°. At the frequencies of 340 GHz and 290 GHz, notable reflectivity is observed from the heads of both the UV resin green and PA12 white honey bees at 175° and 178°, respectively. Furthermore, additional features become visible at different angles that can be attributed to the legs of the honey bees. The RCS plots of the green UV resin honey bee at 770 GHz and 845 GHz, as well as those of the white PA12 honey bee at 685 GHz and 840 GHz frequencies, show numerous narrow deflections, as the resolution for detectable features increases with higher frequencies. The RCS measurements provide multifaceted information, as they help to derive the scattering characteristics of the honey bee model’s body segments. Additionally, the reflections from the body are crucial for achieving accurate imaging. However, the complex surface structure of the bee makes it challenging to comprehend the localized peaks. To validate these RCS measurements, a 2D reconstruction of the bees is performed. Since the RCS does not account for the phase and, consequently, the time-delay of the individual THz-TDS pulses, a further evaluation is imperative. In this case, the 2D reconstruction employs the back-projection algorithm, as demonstrated in^62,63^. Considering the rotation of the honey bees is carried out with fixed antennas, this approach aligns with inverse synthetic aperture radar (ISAR) imaging.

.

**Fig. 13:**
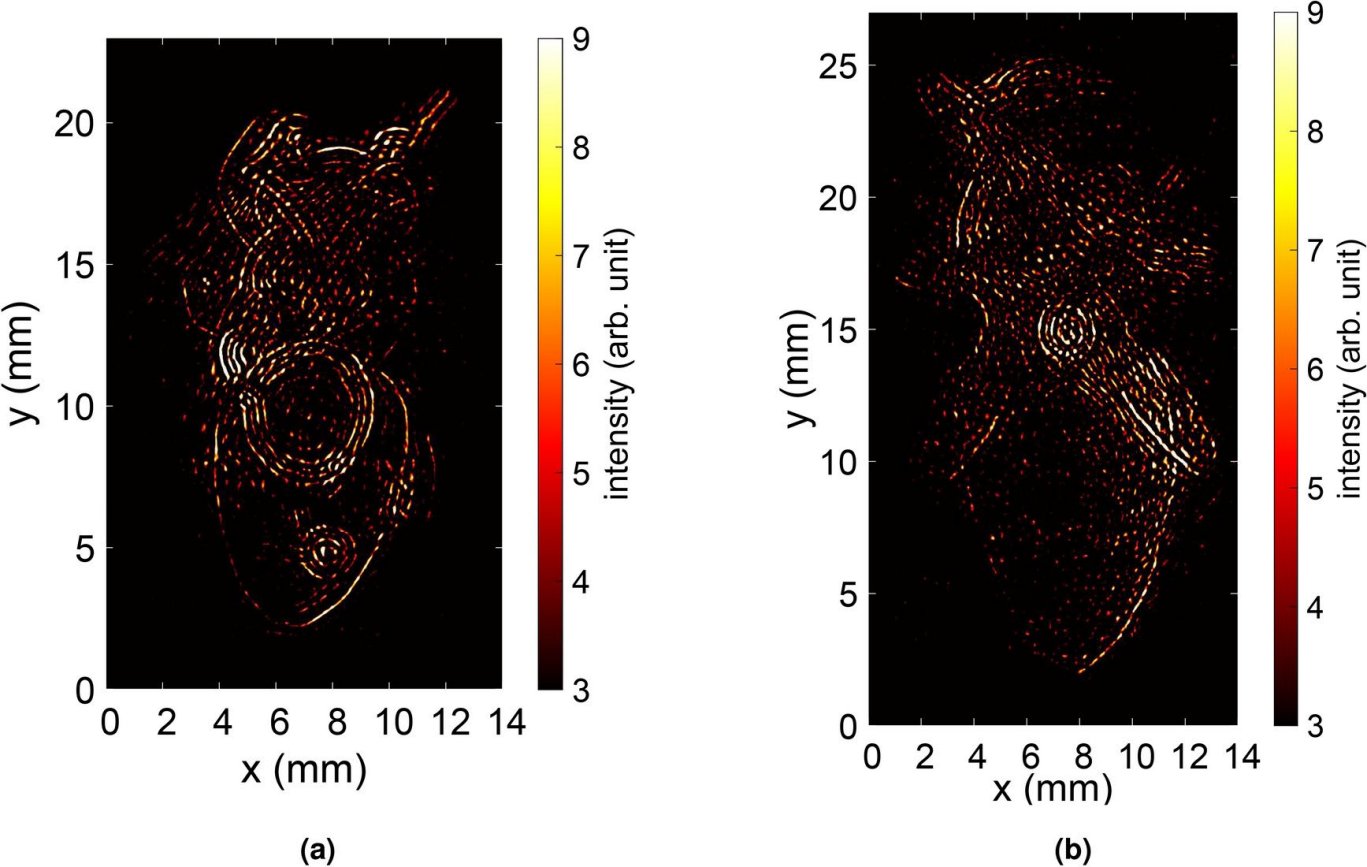
Back-projection of the measured THz-TDS data for (**a**) UV resin green and (**b**) PA12 white honey bee models.

In our previous work, back-projection was performed using divergent Tx and Rx^2,62,63^. In the current study, the emitted radiation is collimated, and the algorithm is adapted to emulate the incoming plane wave for the reconstruction. These reconstructed images are depicted in Fig. 13. For both honey bee models, the body contours are well reconstructed, with different body parts like legs and antennae being conspicuous for both models. Interestingly, the exoskeleton is also very pronounced in the reconstructed images, just as it was in previous work (cf.^2^ Fig. 23). However, unlike measurements with the real honey bee, it cannot be assumed that the shape or water content changes in a way that would alter the electrical material properties. The SAR images reveal that the RCS is primarily dependent on the shape. Hence, honey bee mockups produced by AM can serve as a promising alternative for measurements. Moreover, the gluing interface between the sample holder and the 3D printed bee is visible in the center of the reconstructed images. This aligns with the recorded RCS measurements from Figs. 11 and 12. The spikes in Figs. 11 and 12 highlight the intensity of the reflection, indicating a strong reflection. In general, this work sheds light on the benefits of employing higher frequencies for insect monitoring. The RCS at frequencies beyond 300 GHz is enriched with intricate information about the insects, enabling accurate tracking and identification of small insects at these frequencies.

**Fig. 14:**
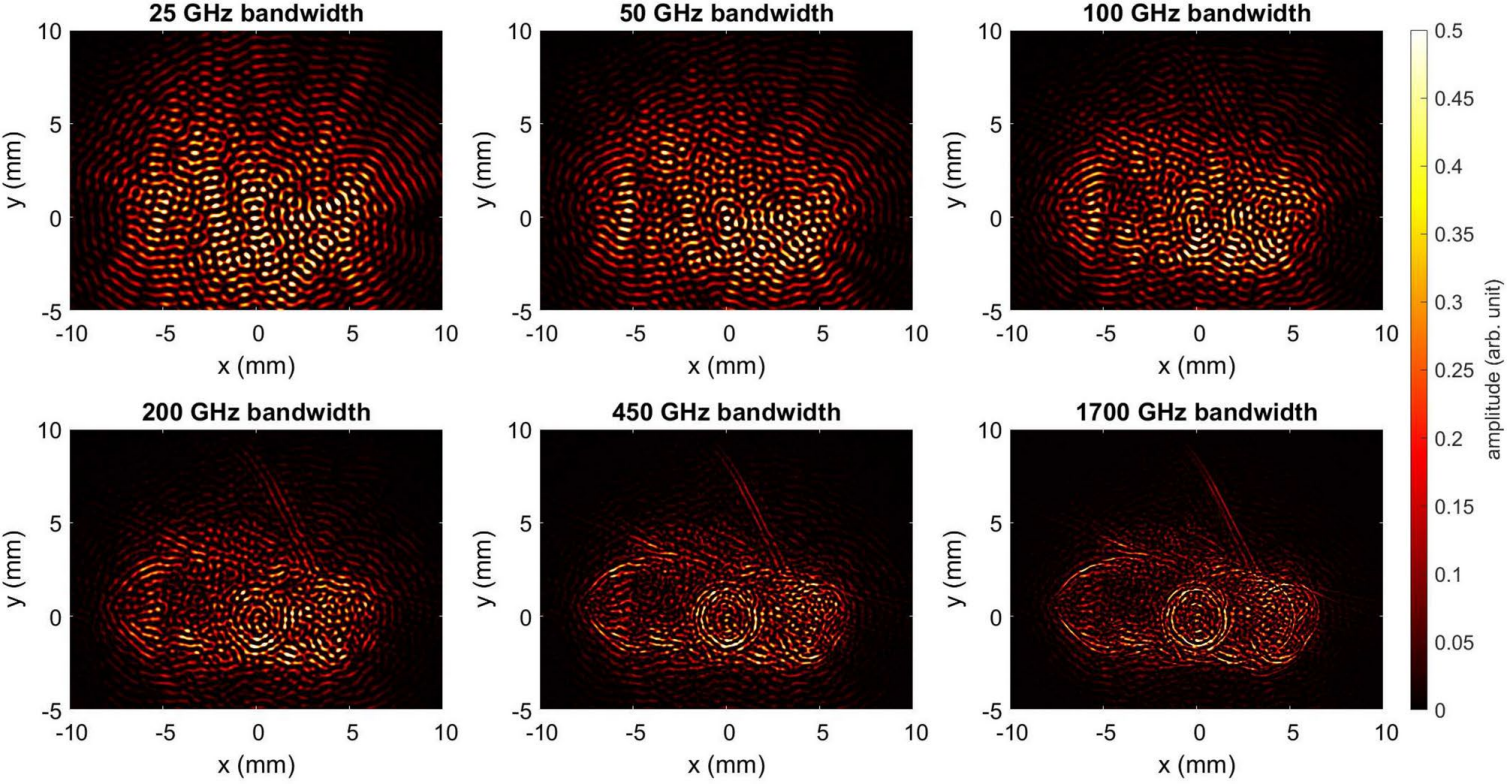
Back-projection of the measured THz-TDS data of a real honey bee. The start frequency for the back-projection is 300 GHz.

**Fig. 15:**
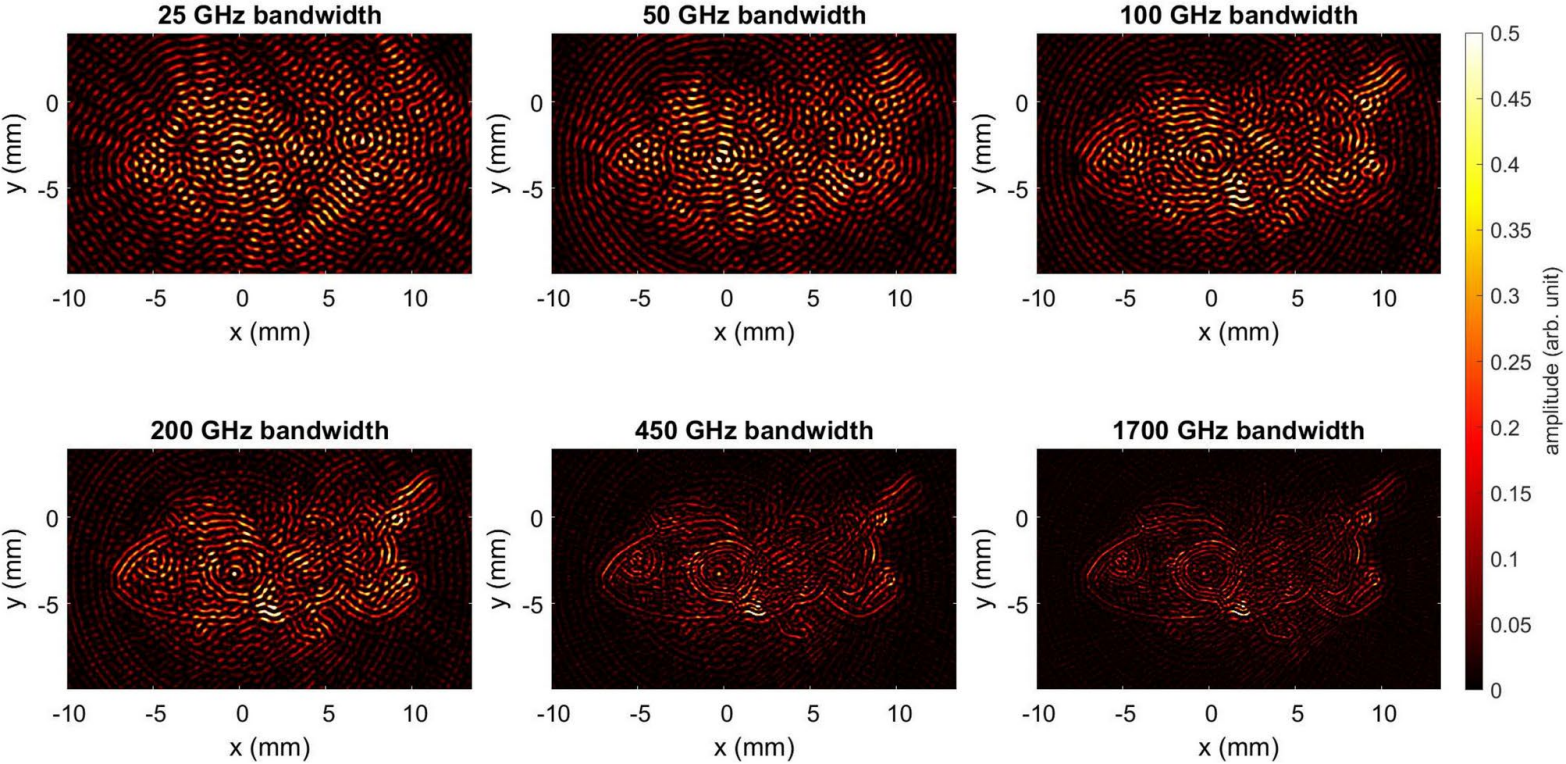
Back-projection of the measured THz-TDS data of 3D-printed green honey bee. The start frequency for the back-projection is 300 GHz.

To validate the scattering behavior of the 3D-printed bee model against that of a real honey bee, a comparison is made by analyzing the back-projection across different bandwidths. The green honey bee model was selected for comparison due to its more realistic anatomy. In our previous work, we conducted measurements on a real honey bee with a comparable setup^2^. The honey bee, mounted on a conical sample holder to reduce unwanted scattering, was collected post-mortem from a beekeeper and preserved in ethanol to prevent dehydration. Using the same TeraK15 system, THz radiation was generated and detected, with a lens to collimate the THz radiation onto the bee. The bee was rotated by 360° in 0.3$^\circ$ steps in azimuth. Figures 14 and 15 present the back-projections for both the real honey bee and the 3D-printed model made from green resin, respectively. These projections are conducted for bandwidths of 25 GHz, 50 GHz, 100 GHz, 200 GHz, 450 GHz, and 1700 GHz, starting from a frequency of 300 GHz. The respective bandwidths range resolutions are calculated using $\Delta r=\frac{c_0}{2B_{\rm w}}$, where $c_0$ is the speed of light in vacuum and $B_{\rm{w}}$ is the bandwidth^64^. For the circular aperture employed, the resulting resolutions are 6 mm, 3 mm, 1.5 mm, 0.75 mm, 0.33 mm, and 0.088 mm, respectively. Due to limited resolution, the bee bodies are not visible at bandwidths of 25 GHz and 50 GHz. With a bandwidth of 100 GHz, the general shape of both the real and 3D-printed bees becomes visible. However, the level of detail remains insufficient to distinguish the bee’s form from other insect shapes. At 200 GHz, the resolution improves, allowing recognition of the overall body structure, as well as features like the antennae (in the 3D-printed model) and parts of the wings (in the real bee). A bandwidth of 450 GHz further enhances contour clarity, providing enough detail to potentially identify the insect type and all body features that reflect THz radiation from the observed angles. While the maximum bandwidth of 1700 GHz sharpens these contours, it does not reveal additional details beyond those at 450 GHz. This aligns with the expected feature sizes along the bee’s body, such as the legs, which have diameters in the range of one millimeter. The bandwidth-dependent back-projection of the real bee and the 3D-printed model exhibits similar behavior, indicating that the 3D-printed bee is a suitable surrogate for imaging purposes. For future studies, 3D-printed bees offer reliable test objects with consistent, well-defined shapes, unlike real honey bees. After testing algorithms, e.g. for insect classification, studies can be extended to real bees. Additionally, examining the frequency-dependent RCS could help identify unique features to differentiate between various insect types. In this study, we explore measurements and simulations obtained from multiple domains to evaluate the interaction between honey bees and EM waves in the THz spectrum. Realistic European honey bee models have been meticulously constructed using advanced modeling techniques in Blender software. Furthermore, these models are then consist of two materials, namely epoxy resin and PA 12. For future applications, concepts for compact photonic THz-TDS systems based on mode-locked laser diodes already exist^65,66^. Recently, it was demonstrated that these systems outperform state-of-the-art systems in terms of signal-to-noise ratio in the frequency range below 500 GHz despite their compact design^67^.

**Fig. 16:**
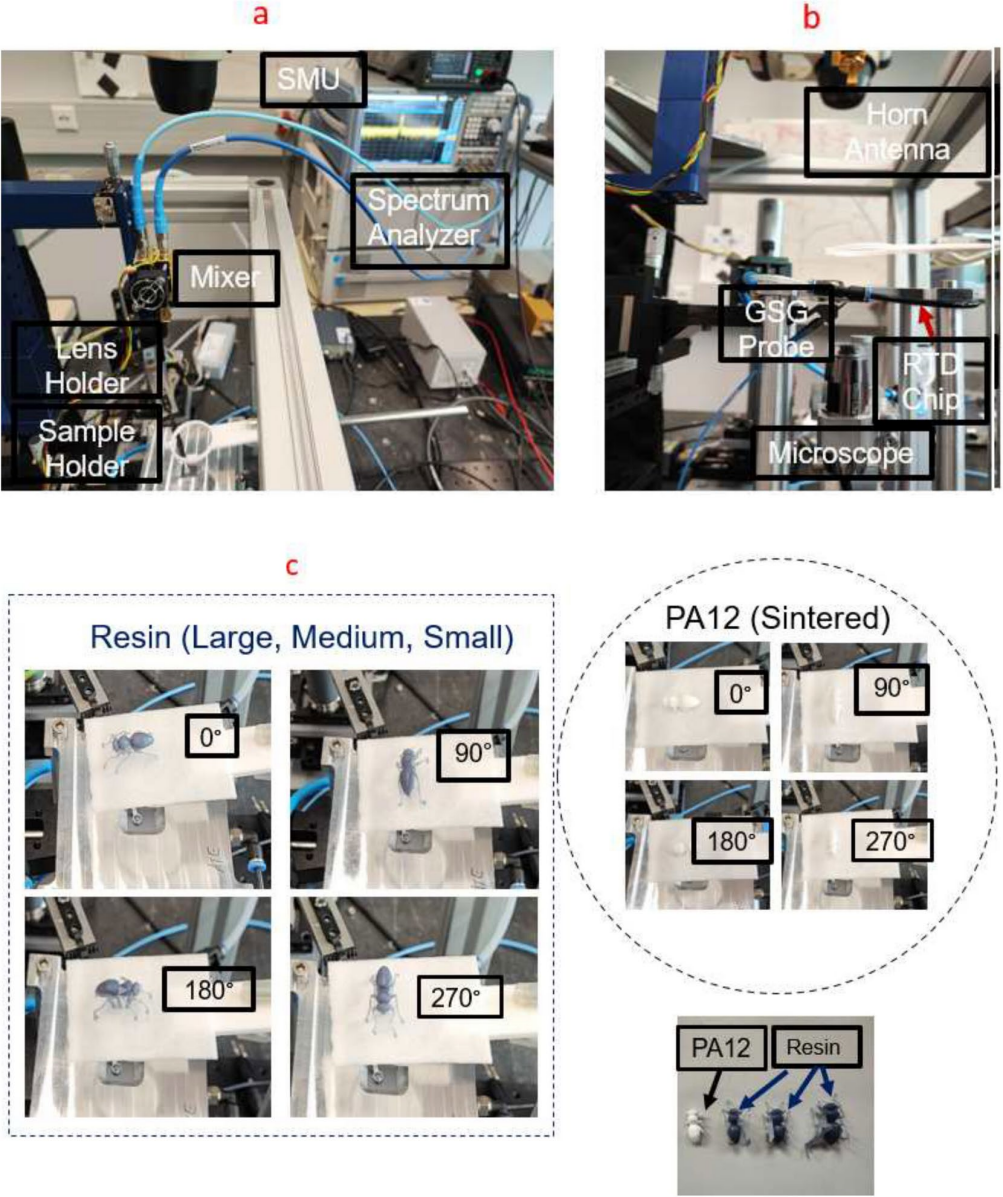
Measurement setup for the scattered power measurements featuring RTD Oscillator chip integrated with slot antenna, and the connection of a spectrum analyzer to the WR3.4 Mixer from VDI for power recording. (**a**) top view (**b**) side view, and (**c**) positioning of 3D UV resin and powder bed-fused PA12 honey bee models during rotations at 0°, 90°, 180°, 270°. The four chosen samples comprise realistic 3D UV resin honey bee models in small, medium, and large sizes, accompanied by a powder bed-fused PA12 realistic 3D honey bee model.

*Frequency domain measurements with RTD THz source*. In this study, we experimentally investigate the interaction of EM radiation with AM-generated models of European honey bees at frequencies around 250 GHz. Previous studies on this interaction among honey bees relied solely on creating highly accurate 3D digital models of the honey bees to assess radiation exposure along with scattering analysis. It is noteworthy to mention that the electrical properties of the insects are incorporated into these models, accounting for the random surface roughness of the insects when exposed to THz frequencies, as their bodies appear rough to radiation at these frequencies. The orientation of the honey bee is a determining factor for the scattered power emanating from its different parts. The variations between honey bee types and sizes is attributed to surface scattering. Orientation-dependent measurements are conducted to analyze the scattering of EM waves for bees at different azimuthal angles, providing valuable data for RCS analysis. The experimental setup utilized in this study for THz scattered power measurements is illustrated in Fig. 16a. This setup comprises several crucial components: the RTD oscillator chip, featuring its integrated on-chip antenna, secured in place using a custom-designed vacuum chuck positioned upside-down for the measurements. The radiated signal is efficiently extracted from the oscillator through a hyper-hemispheric high-resistivity float-zone silicon (HRFZ-Si) 5 mm diameter lens placed on the backside of the chip, DC Biasing is achieved by enabling electrical contacts with the GSG (ground-signal-ground) probe on the chip’s front side.

The GSG probe is connected to a source-measure unit (SMU) by a coaxial mm-wave cable to prevent spurious oscillation. THz radiation is received using a WR3.4 diagonal horn antenna with a 25 dBi gain and an effective area of 8.8 mm^2^, connected to a Heterodyne mixer (WR3.4MixAMC-I, Virginia Diodes Inc). Measurements are recorded with a phase noise analyzer (FSWP50, Rohde & Schwarz) in spectrum analysis mode. This setup enables for precise measurement and analysis of the RF signal characteristics. The horn antenna’s opening was positioned 18.5 cm away from the oscillator in the far field, resulting in a free-space path loss of 63.2 dB. We utilized additively manufactured polylactide (PLA) holders to maintain alignment of the lens and secure the different honey bee models. Precise alignment among the lens, oscillator, and horn antenna was crucial for the measurements. Before contacting the RTD chip with the GSG probe, optical alignment of the lens holder and the horn antenna was performed using a microscope. Fine-tuning of the alignment was achieved through micrometer screws to control the relative movement of the lens and chip. When evaluating the offset between the mixer and the lens, any placement errors were compensated by adjusting the lens in the opposite direction. Through an iterative process, the positions of the lens and the horn antenna were fine-tuned, respectively, while maximizing the measured received power.

**Fig. 17:**
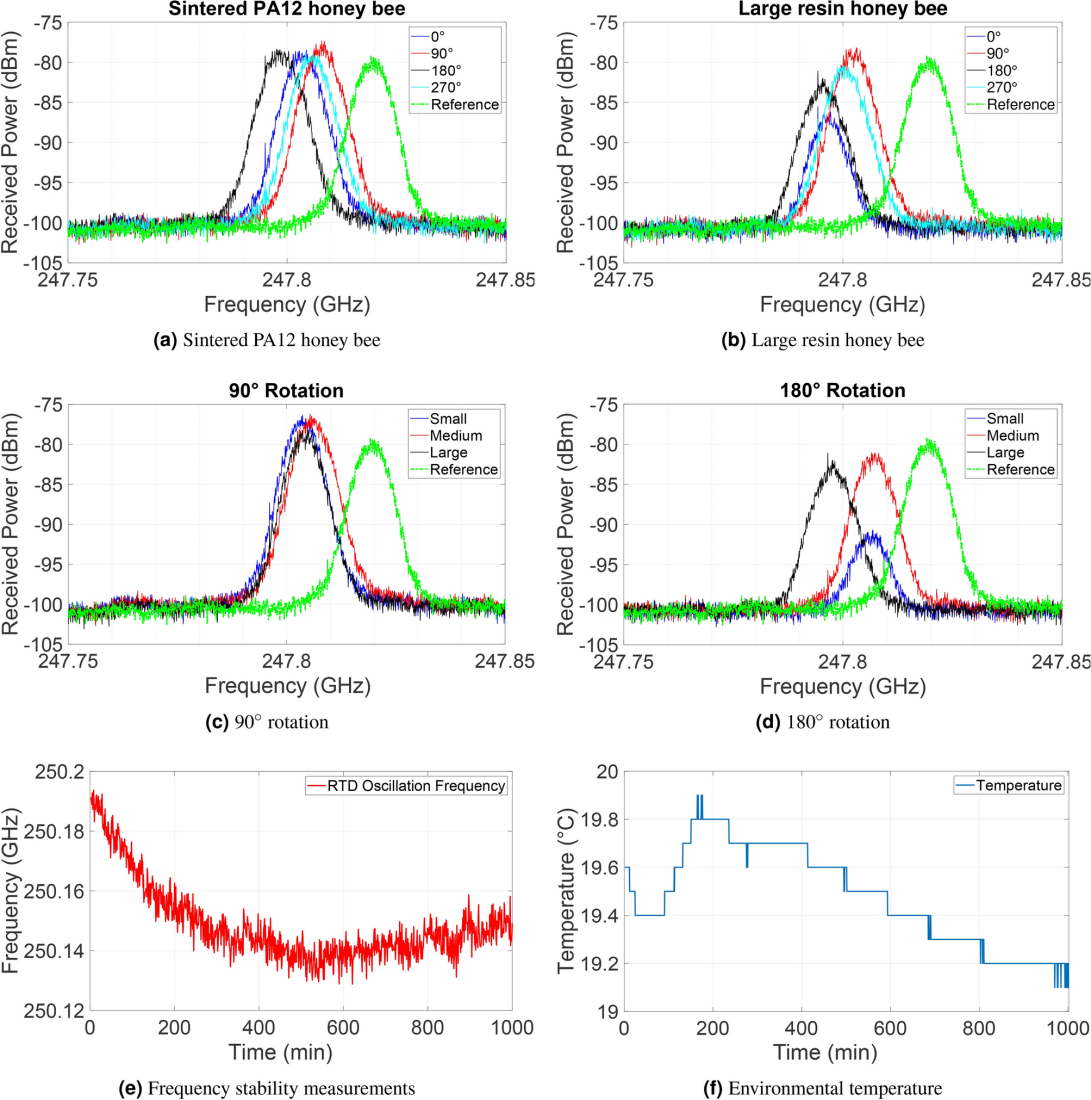
The recorded power spectra from the spectrum analyzer for two different samples and four different rotations: (**a**) powder bed-fused PA12 honey bee, (**b**) large resin honey bee. The recorded power spectra from the spectrum analyzer for the 3D constructed model of honey bee made by resin with different sizes small, medium, and large for different rotations: (**c**) 90°, (**d**) 180°. (**e**) Frequency stability measurements of RTD oscillator over time. (**f**) The environment’s temperature recorded with the data logger.

Figure 16 illustrates the measurement setup in detail. A bias point of -0.47 V in the NDR region of the RTD is selected for all sample measurements. Four insect samples, as depicted in Fig. 16c, are investigated. These samples consist of a realistic 3D UV resin honey bee model in three different sizes (small, medium, and large) along with a powder bed-fused PA12 realistic 3D honey bee model mimicking insect dielectric properties. Each sample is positioned midway between the RTD oscillator chip and the mixer’s horn antenna, following the arrangement shown in Fig. 16c. The design of the sample holder is crucial to minimize interference and ensure that RCS measurements primarily reflect the scattering properties of the bee alone. Each sample undergoes individual 90° steps of rotation in the azimuth plane until completing a full 360° rotation. The scattered radiation is measured for each rotational position. For each measurement, a total of 70 traces, each with 10,001 points in frequency, is collected and then averaged to obtain accurate power data. Once the main oscillation peak in the 225–262 GHz frequency range is discovered, the power density of the spectrum analyzer is symmetrically integrated for all frequencies within the 600 MHz band, thus subtracting the noise floor. In order to study the influence of the honey bee model on the frequency and scattered power, a reference measurement using a blank sample holder is recorded. This reference measurement helps establish a known value, providing insight into the impact of various honey bee sizes and rotational positions on the measurements. Further details are discussed in the following passage.

Figure 17 illustrates the recorded power spectrum around the peak frequency for different measured samples. For clarity purposes, only the 100 MHz band around the peak is presented in these plots. In the case of the powder bed-fused PA12 honey bee, the peak values are almost the same for all rotations, as shown in Fig. 17a. This can be attributed to the lack of intricate details in the insect body, like wings and legs. Furthermore, in Fig. 17a, it is evident that the peak power frequency exhibits a behavior almost identical to the previous measurements on the PA12 sample, with a shift to lower frequencies compared to the reference. This effect can be attributed to the detuning of the main frequency of the RTD oscillator in the presence of these dielectric honey bee samples, a phenomenon observed consistently in all four measurements. As for the UV resin honey bee, changes in the scattered power are observed for each 90° step rotation in the case of the large sample, as shown in Fig. 17b. Upon comparison, it can be seen that these scattered power measurements are several dB higher for the vertical rotations (90° and 270°) compared to the horizontal rotations (0° and 180°), corroborating findings from prior research on measured real honey bees using THz-TDS^2^. Examining Fig. 17c,d, the impact of sample size on received power is illustrated for different rotations. For vertical rotations (90° and 270°), power values show negligible change, remaining nearly constant across different sample sizes. Conversely, during horizontal rotation (0° and 180°), considerable changes in values occur with alterations in sample size. To elaborate, at the same rotation angle in horizontal rotation, the medium and large honey bee samples exhibit higher reflected power (several dB) than the small sample. This observation could reasonably be attributed to the RCS of the honey bee. Upon comparing the amplitude of the scattered power during both vertical and horizontal rotations, it is apparent that vertical rotation yields higher received power from the sample compared to horizontal orientation. This phenomenon can be explained as follows: when the samples are placed in the vertical position, the honey bee model enhances the directivity of the radiated beam from the RTD oscillator. Additionally, in the presence of the dielectric PA12 honey bee sample, the natural frequency of the RTD oscillator is detuned, resulting in a slight shift in frequency and an increase in power. Consequently, we anticipate a change in RTD output power when altering the impedance of the oscillator tank, including the antenna and surrounding dielectric material, as perceived by the RTD.

It is noteworthy to mention that the rough surface of the model can induce variations in incident, reflection, and scattering angles across its body, resulting in stronger backscatter power from the model at certain rotational positions. The determination of bee orientation by polarized THz radiation can be linked to a prior study on real honey bees, demonstrating that the outer shell of the honey bee significantly reflects energy, allowing for to the observation of the three main body parts: head, thorax, and abdomen. It was observed that the reflected energy from the rear part of the bee’s body is relatively lower compared to the front, with a difference of approximately 10 dB^2^. To investigate quantitatively the stability of our fabricated RTD oscillator, we conducted free-running spectral measurements for up to 16 hours, simultaneously monitoring the temperature of the laboratory with an environmental data logger (Testo). Figure 17e,f display the oscillation frequency of the RTD oscillator alongside measurements of the environment’s temperature. The calculated drift in frequency during the time measurement period was 0.2 MHz/min for the free-running RTD oscillator and approximately 5.4–10.1 MHz/min for different honey bee sample measurements. Therefore, it can be inferred that bee orientation caused a significant change in the frequency.

## Discussion

We investigated various measurement and simulation directions to evaluate the interaction between honey bees and EM waves in the THz spectrum. Realistic European honey bee models, meticulously constructed using advanced modeling techniques in Blender software, significantly contributed to being additively manufactured with two materials, namely epoxy resin and PA12. The AM-generated models played a pivotal role in all measurement campaigns. In this work, we demonstrated a change in THz response for scattered propagation measurements with different bee-mimicking samples using an RTD as a THz source. A correlation between bee orientation and THz wave polarization could be inferred from frequency shifts and changes in amplitude, depending on the bee model. These results highlight the capability to gather detailed information about insect morphology across different monitoring environments, like beehives, allowing for the differentiation of various types and sizes of honey bees. RTD THz sources, known for being low-cost, compact, robust, and power-efficient, could facilitate distributed wireless, non-invasive, continuous monitoring networks for insect observation.

The results provided by THz-TDS emphasize not only the optional opportunity for insect monitoring with THz technology but also the necessity to continue following this research path owing to its advantages for accurate tracking and identification of small insects. Typically, the sub-wavelength resolution may outperform sub-THz and mmWave systems and enable the contactless monitoring of smaller insects. Obviously, the bee models printed by AM provide only an approximation to the real electrical material parameters. Nevertheless, their properties can be evaluated as constant, enabling repeatable measurements, detecting RCS fingerprints, and supporting the reconstruction of pictures by ISAR. As a validating approach, the scattering behavior of honey bee mockups and real bees exhibits similarities. Furthermore, a minimum bandwidth of 100 GHz is required to identify the bee’s contours through imaging. However, a bandwidth of 450 GHz sharpens the resulting image, providing a more pronounced depiction of the bee’s shape due to the improved resolution.

A notable aspect of this study is the revelation that honey bees exhibit heterogeneity. The detailed and extensive material characterization is crucial for anticipating EM-induced stress of honey bees within the THz region. As a result, this material characterization enhances the accuracy of full-wave computational EM simulations. The RF-EMF exposure of a honey bee worker was estimated at 300 GHz using numerical simulations. These simulations reveal that at very short distances (1 mm) from the antenna, the bee can absorb up to 26% of the input power into an antenna. In the near-field, the majority of the power is absorbed in the bee’s exoskeleton. However, in the far-field, the inner tissues absorb a slightly higher relative fraction of the absorbed power. Observing compliance with the ICNIRP reference levels in the THz application, the maximally allowed far-field exposure of 10 W/m^2^ corresponds to an average absorbed power of 0.29 mW into a honey bee worker.

### Future work

Undoubtedly, this presented work is paving the way to gain a better understanding of honey bee fingerprints derived from both simulation and measurements. Monitoring an important pollinator type, specifically the European honey bee, represents progress towards continuous insect monitoring. However, future research needs to consider various other aspects, such as multi-insect monitoring, insect-insect, insect-plant, and insect-human interactions, particularly in the case of pollinators and pests. Nevertheless, this topic has the potential to monitor complex biotopes in dynamically changing states, particularly owing to the consequences of climate change. Tracking pollinators and pests is of vital importance to derive information related to agricultural interests or to halt the spread of tropical diseases. Field tests and digital twins that closely mimic typical biotopes are targeted for future evaluation of the reliability of THz technologies.

## Data Availability

The data that support the findings of this study are available from the corresponding authors upon reasonable request.
